# Deciphering Key Regulatory Roles of Linear Ubiquitination in Cell Fate Determination and Disease

**DOI:** 10.34133/research.1061

**Published:** 2025-12-23

**Authors:** Jianjia Huang, Linlin Tao, Jianzhao Liao

**Affiliations:** ^1^ Laboratory Animal Center, Fudan University, Shanghai, P.R. China.; ^2^ Department of Veterinary Internal Medicine, College of Veterinary Medicine, South China Agricultural University, Guangzhou, P.R. China.

## Abstract

Cell fate determination is a complex and dynamic process. Across diverse cellular contexts, various protein modifications (PTMs) are involved throughout the entire process of cell fate determination. Linear ubiquitination is a specific type of PTM. Under the catalysis of linear ubiquitin chain assembly complex, multiple ubiquitin molecules form a unique linear ubiquitin chain and modify the substrate. A growing number of studies have shown that linear ubiquitination is an important regulatory target in the cell fate determination pathway, and that once the linear ubiquitination process of key proteins is defective, the cell fate determination process will be disrupted and lead to the development of various diseases. In this review, we systematically elaborated the key links between linear ubiquitination and cell fate determination from the aspects of cell proliferation, differentiation, senescence, and survival/death. Furthermore, we provide an in-depth exploration of the molecular mechanisms through which homeostatic imbalance of linear ubiquitination contributes to numerous diseases, including neoplastic, neurodegenerative, infectious, inflammatory, and metabolic disorders. Finally, we fully affirm the significance of linear ubiquitination as a promising therapeutic target for future drug discovery and development. Our critical appraisal and synthesis of the literature provide a foundational framework for future investigations and inform novel strategies for the treatment and management of related diseases.

## Introduction

Protein modifications, also referred to as post-translational modifications (PTMs), are chemical modifications that occur to proteins after biosynthesis. These modifications include the chemical changes of amino acids (e.g., citrullination), the addition of groups (e.g., phosphorylation, glycosylation, acetylation, and methylation), and the covalent coupling of small peptides or proteins (e.g., neddylation, sumoylation, and ubiquitination) [1,2]. PTM is essential for various biological processes and indispensable for life since proteins require appropriate folding and PTM to function normally [3].

Ubiquitin is a 76-amino-acid polypeptide that is covalently attached to specific amino acid residues within substrate proteins. This PTM, known as ubiquitination, can further modulate protein function and homeostasis by influencing protein folding, degradation, and protein–protein interactions [4]. Ubiquitination controls multiple physiological processes in cells, including proteasome-mediated degradation, DNA damage repair, and the cell cycle [4]. The ubiquitination cascade reaction requires 3 mutually synergistic enzymes: the E1 ubiquitin-activating enzyme, the E2 ubiquitin-coupling enzyme, and the E3 ubiquitin ligase. Only with the help of these 3 enzymes can ubiquitin be combined with its substrate smoothly [5]. Ubiquitination can be classified into multiple distinct types based on the architecture of the ubiquitin chain, the linkage modes between ubiquitin molecules, and the number of ubiquitin molecules conjugated to the substrate. Notably, linear ubiquitination represents a highly distinctive form of ubiquitin modification. When the N-terminal methionine (Met1) residue in ubiquitin forms a peptide bond with the carboxyl group at the C-terminal Gly residue of another ubiquitin, this generates an “end-to-end” linear ubiquitin chain with Met1 as the linkage site [6]. Unlike other forms of ubiquitination that use Lys residues for coupling, this unique linear ubiquitination has been shown to play a key role in cell proliferation, cell survival/death, immune response, and bacterial clearance through selective autophagy [7].

Cell fate determination is the process and its outcome by which a cell adopts a specific identity or functional state during development or in response to environmental cues. Key processes in cell biology, such as cell proliferation, differentiation, senescence, survival, and death, are central manifestations of this decision-making process. This process is determined by the gene regulatory network, signal pathway, and external environment [8]. Once the regulation of cell fate is hindered, it leads to a series of serious consequences, such as cancer. The ubiquitination system has been shown to be an important decision-maker in cell fate, influencing the direction of cell fate through intervention at multiple levels [9]. Therefore, further study on the relationship between ubiquitin-dependent modifications and cell fate is of crucial significance for understanding the molecular mechanisms of cell fate determination and the pathogenesis of the related diseases. This review focuses on the key mechanisms by which linear ubiquitination governs cell fate determination and the association between its dysregulation and various diseases. Its aim is to provide a comprehensive and critical synthesis of the field, thereby offering essential references and novel perspectives to guide future research, therapeutic strategies, and drug development.

## Overview of Linear Ubiquitination

### Linear ubiquitin chain assembly complex

The linear ubiquitin chain linked via Met1 is the only non-Lys residue-dependent chain linkage and is therefore biochemically and structurally unique. Up to now, the only ligase complex known to produce a linear ubiquitin chain is the linear ubiquitin chain assembly complex (LUBAC) [10]. LUBAC consists of 3 subunits: heme-oxidized IRP2 ubiquitin ligase 1L (HOIL-1L), HOIL-1-interacting protein (HOIP), and shank-associated RH domain-interacting protein (SHARPIN) [11].

HOIL-1L belongs to the RING-between-RING (RBR) ubiquitin ligase family. It is an E3 ligase that catalyzes the formation of ester bonds between the C-terminal carboxylic group of ubiquitin and Ser or Thr residues of its substrates (including 3 subunits of LUBAC), subsequently initiating the de novo synthesis of the ubiquitin chain [12]. HOIL-1L couples monoubiquitin to all subunits of LUBAC via its RBR domain and causes HOIP-mediated binding of linear ubiquitin chains to monoubiquitin (i.e., auto-linear ubiquitination of LUBAC), which inhibits LUBAC function [13]. On the other hand, HOIL-1L interacts with HOIP through the ubiquitin-like (UBL) domain, promoting the stimulation of HOIP activity [14]. There is a unique interaction pattern between HOIL-1L and SHARPIN, mediated by LUBAC-tethering motifs (LTMs) that fold into a single spherical domain; the dimerization mediated by LTM plays a decisive role in the stability of the LUBAC [15].

HOIP belongs to the RBR E3 ubiquitin ligase family, and its RBR domain is the catalytic center for linear ubiquitin [16]. The RBR domain contains an N-terminal RING1 and a C-terminal RING2, and the 2 domains are connected by the in-between-RING (IBR) domain [17]. HOIP recognizes and chelates the ubiquitin-carrying E2 enzyme at RING1 and transfers ubiquitin from E2 to specific Cys residues in RING2 (Cys885 in human), instantaneously forming a homologous to E6-associated protein C-terminus (HECT)-like covalent thioester intermediate. At this point, at the C-terminus of HOIP, the linear ubiquitin chain determining domain (LDD), which interacts with the receptor ubiquitin, promotes the covalent connection of the C-terminus of the donor ubiquitin to the N-terminus of receptor ubiquitin, and ultimately mediates the formation of the linear ubiquitin chain [16,18]. Due to the intramolecular interaction between the RBR domain and its N-terminal domain, HOIP exists in an auto-inhibited state, but when HOIP interacts with the UBL domain in SHARPIN and HOIL-1 through its ubiquitin-associated domain 1 and 2 (UBA1 and UBA2) respectively, HOIP is able to release its auto-inhibition and exerts its function as E3 ligase [14,15]. The peptide:*N*-glycanase and UBA or ubiquitin regulatory X (UBX)-containing proteins (PUB) domain of HOIP primarily mediates interactions with OTU deubiquitinase with linear linkage specificity (OTULIN) and other binding partners [19]. HOIP also contains a B-box domain, but its exact functional mechanism is not fully understood at present.

SHARPIN is a protein consisting of 387 amino acids and the last subunit characterized in LUBAC. In addition to LTM, SHARPIN is currently known to contain pleckstrin homology (PH), UBL, and Npl4 zinc finger (NZF) domains: the N-terminal PH domain mediates homodimerization and is associated with β1 integrin and tumor regulation [20]; the UBL domain interacts with the UBA1 domain of HOIP to mediate the formation of LUBAC [14]; and the C-terminal NZF domain possesses the function of recognizing and binding ubiquitin and prevents tumor necrosis factor alpha (TNF-α)-induced cell death [21], but so far, the full-length structure of SHARPIN, as well as the specific structure of NZF, has not been completely analyzed. SHARPIN itself cannot produce linear ubiquitin modification, but it can activate the activity of LUBAC when bound to HOIP [22]. It has been shown that SHARPIN can replace HOIL-1L to facilitate HOIP to synthesize linear ubiquitin chains [20]. In addition, endogenous HOIP can form dimers with only HOIL-1L or SHARPIN in the absence of HOIL-1L or SHARPIN, and either dimer can synthesize a linear ubiquitin chain to activate nuclear factor-κB (NF-κB) [20].

Meanwhile, all 3 subunits of LUBAC contain ubiquitin-binding domains (UBDs), such as the ZF and/or NZF. These UBDs play an important role in the process of LUBAC recruitment into receptor-related signal complexes [16]. In summary, the stability of LUBAC complex is determined by the interaction among HOIP, HOIL-1L, and SHARPIN. The 3 subunits are combined in a stoichiometric ratio of 1:1:1 to maintain the function of LUBAC, but the exact number of 3 subunits in the complex is still unknown [10], and the loss of any component of LUBAC will reduce the stability of other components and diminish the activity of LUBAC [16].

### Deubiquitinating enzymes that remove linear ubiquitin chains

Linear ubiquitination modification is a dynamic and reversible process under strict control. This reversibility is achieved by deubiquitinating enzymes (DUBs) that specifically cleave linear ubiquitin chains. There are only 2 kinds of DUBs known to be capable of removing linear ubiquitination, namely, OTULIN and cylindromatosis (CYLD). OTULIN is the only DUB so far shown to specifically cleave linear ubiquitin chains [19], and CYLD cleaves K63-linked polyubiquitin chains in addition to Met1-linked linear ubiquitin chains, thereby modifying the substrate protein via deubiquitination [23].

OTULIN has a highly conserved OTU domain at the C-terminus, which is noted to be characteristic of the papain-like family of Cys peptidases [19]. The N-terminus of OTULIN contains a PUB-interacting motif (PIM) that mediates direct interaction with the N-terminal PUB domain of HOIP [19]. The interaction between OTULIN and HOIP is regulated by multiple factors, and the binding of OTULIN to HOIP is blocked when Tyr56 on PIM is phosphorylated [16]. When a specific E3 ligase-tripartite motif-containing protein 32 couples polyubiquitin linked by K63 at 2 Lys residues (Lys64 and Lys66) near the PIM domain, it inhibits the interaction between OTULIN and HOIP and promote the activation of NF-κB induced by TNF-α [19]. The study confirmed that OTULIN is a key player in the regulation of linear ubiquitination of intracellular proteins [24]. Inactivation of OTULIN results in a severe disruption of linear ubiquitin chain homeostasis within cells [24]. In the normal state, OTULIN cleaves the linear ubiquitin chain on LUBAC subunits, thus derepressing the self-inhibition of LUBAC [16]. OTULIN appears to have a specific ubiquitin-binding site for the Met1 linkage, which enables it to interact specifically with linear chains, and in the presence of both the K63 and Met1 linkages, this ubiquitin-binding site allows OTULIN to interact almost exclusively with the Met1 linkages without binding other ubiquitin chain types [25]. OTULIN possesses a mechanism of substrate-assisted catalysis that enables it to distinguish and hydrolyze Met1 linkages specifically [25]. Regardless of where the linear chains are attached, this mechanism appears to enable OTULIN to selectively bind Met1-linked ubiquitin chains with high specificity rather than to target individual proteins [25].

**Fig. 1.**
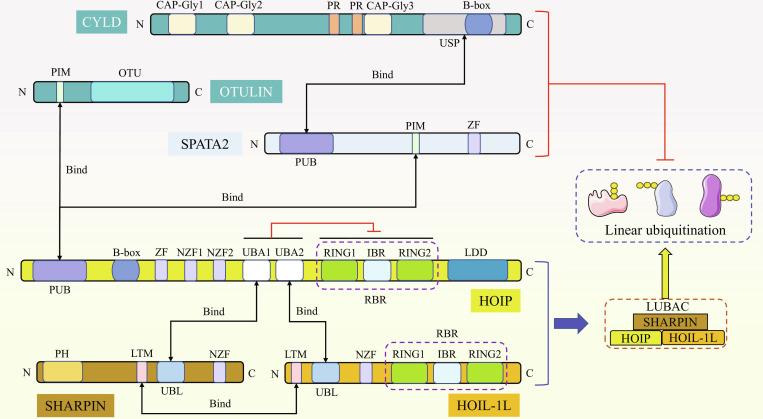
Domain structure of LUBAC subunits, OTULIN, CYLD, SPATA2, and their respective interactions. LUBAC consists of 3 subunits, HOIP, HOIL-1L, and SHARPIN. The RBR domain at the C-terminus of HOIP is the linear ubiquitin catalytic center of LUBAC. HOIP contains LDD, UBA1/2, NZF1/2, ZF, B-box, and PUB domains, and it interacts with the UBL domains of SHARPIN and HOIL-1L through the UBA1/2 domain. HOIL-1L possesses LTM and NZF domains like SHARPIN in addition to the UBL and RBR domains, and the two interact through the LTM domain. Additionally, SHARPIN contains a special PH domain that mainly mediates homodimerization. OTULIN contains a conserved OTU domain and a PIM domain that interacts with the PUB domain of HOIP. CYLD contains CAP-Gly1/2/3, PR, and USP domains, with the latter specifically encompassing the B-box domain. SPATA2 interacts with the USP domain of CYLD through the PUB domain and then selectively binds to the PUB domain of HOIP through the PIM domain, and the ZF domain is also present in SPATA2.

CYLD belongs to a ubiquitin-specific protease (USP) family, and it consists of 3 glycine-rich cytoskeleton-associated protein (CAP-Gly) domains, 2 proline-rich motifs (PR), a phosphorylation region, and an N-terminal USP catalytic domain. The zinc-finger-like B-box domain is contained within the USP domain, which mediates the subcellular localization of CYLD [26]. In contrast to the direct interaction of OTULIN with HOIP, CYLD interacts with the PUB domain of HOIP indirectly, requiring bridging through spermatogenesis-associated 2 (SPATA2). SPATA2 contains PUB, PIM, and ZF domains, and SPATA2 selectively binds to the PUB domain of HOIP through the PIM domain but does not recognize other PUB domains, while SPATA2 activates CYLD by interacting with the USP domain of CYLD through the PUB domain in a PIM-independent manner [27]. In addition, CYLD homodimerizes via its B-box domain and subsequently associates with 2 SPATA2 proteins to form a heterotetramer [28]. Since both SPATA2 and OTULIN interact with the PUB domain of HOIP and recognize the same binding motif, SPATA2/CYLD and OTULIN compete with each other during the interaction with HOIP, indicating that SPATA2 prevents excessive cleavage of linear ubiquitin chains on LUBAC by OTULIN [16]. In contrast to OTULIN, CYLD has the ability to hydrolyze both K63- and Met1-linked ubiquitin chains, though it shows a preference for linear chain removal in vitro [29]. Figure 1 shows the key domains and interaction modes of OTULIN, CYLD, SPATA2, and LUBAC subunits.

## Linear Ubiquitination: Roles in Cell Proliferation and Differentiation

### Overview of cell proliferation and differentiation

Cell proliferation is the process through which cells divide and multiply, leading to an increase in cell number. This highly regulated process involves key events like DNA replication and is controlled by cell cycle regulatory factors. The intricate regulatory network among cyclins, cyclin-dependent kinases (CDKs), and CDK inhibitors constitutes a fundamental mechanism that ensures the orderly progression of the complete cell cycle [30]. Conversely, cell differentiation, the process by which unspecialized cells acquire specific identities and functions, underpins the development of an organism. The formation of complex tissues and organs is the ultimate manifestation of this process. It has been established that the control of cell proliferation is associated with cell differentiation, which serves as a critical mechanism for inhibiting cell proliferation under physiological conditions [31]. Cell proliferation and differentiation are pivotal processes in tumorigenesis. Benign tumors typically exhibit dysregulated proliferation but retain a degree of differentiation comparable to normal cells. In contrast, malignant tumors are characterized by both uncontrolled proliferation and aberrant differentiation [32,33]. Therefore, maintaining a balance between proliferation and differentiation is crucial for normal development and tissue homeostasis in multicellular organisms.

### Linear ubiquitination mediates cell proliferation and differentiation

LUBAC primarily regulates the proliferation and differentiation of immune cells, with B lymphocytes and T lymphocytes showing a strong association with linear ubiquitination. B lymphocytes are a vital component of the immune system, and LUBAC-dependent NF-κB activation is essential for B-cell development and function. It has been demonstrated that LUBAC deficiency impairs transmembrane activator and calcium-modulating cyclophilin ligand interactor (TACI)-, CD40-, and Toll-like receptor 4 (TLR4)-mediated NF-κB activation by inhibiting inhibitor of κB (IκB) kinase (IKK) complex activation, ultimately leading to impaired B1 cell development in mice [34]. Notably, B-cell receptor (BCR)-induced NF-κB activation remains unaffected by LUBAC deficiency [34]. Furthermore, deficiency in LUBAC activity not only impairs the proliferation and differentiation of B1 cells in mice but also triggers extensive TLR4-mediated B-cell death. In addition, HOIP-deficient mice exhibit a severely attenuated antibody response upon lipopolysaccharide (LPS) stimulation [35]. These findings clearly demonstrate that the linear ubiquitination activity of LUBAC is essential for the normal proliferation, differentiation, and survival of B1 cells. Consistent with findings in mice, HOIP-deficient patients exhibit marked defects in CD40-dependent B-cell activation and plasmablast differentiation [36]. This evidence underscores the indispensable role of LUBAC activity in human B-cell development.

T lymphocytes originate in the bone marrow and mature in the thymus. The critical importance of LUBAC activity for the survival and development of mature T cells has been well established. Loss of LUBAC function in mice severely impairs the development of natural killer T (NKT) cells, as well as the proliferation and differentiation of CD4^+^ T cells [37]. NF-κB activation is a prerequisite for the normal proliferation and differentiation of both B lymphocytes and T lymphocytes. In T cells, the T-cell receptor (TCR)–NF-κB pathway is predominantly regulated by LUBAC. Deficiency in LUBAC prevents TCR stimulation from activating NF-κB via NF-κB essential modulator (NEMO), ultimately leading to impaired proliferation and developmental defects in CD4^+^ T cells in mice [37]. In Jurkat cells, TCR stimulation promotes the deposition of linear ubiquitin chains by LUBAC onto the CARD11–BCL10–MALT1 (CBM) complex. This linear ubiquitination subsequently facilitates the stable activation of the IKK complex and downstream NF-κB signaling. However, this process is specifically inhibited by OTULIN, but not by CYLD [38]. The authors indicate that the linear ubiquitination of the CBM complex promotes the subsequent activation of NEMO within the IKK complex in a manner that is independent of further LUBAC involvement. This pathway represents the primary mechanism for TCR–NF-κB pathway activation in Jurkat cells [38]. Furthermore, LUBAC activity is essential for the differentiation of Forkhead box-P3^+^ regulatory T cells (Tregs) and NKT cells in the murine thymus, as well as for the maintenance of peripheral Tregs [39]. Thymic epithelial cells (TECs) play a vital role in orchestrating T-cell differentiation and establishing immune tolerance. Maintaining LUBAC-mediated linear ubiquitination is not only a prerequisite for the survival and differentiation of TECs but also a critical requirement for normal thymic development and the establishment of a naive T-cell repertoire in mice [40].

In addition to B and T lymphocytes, the development of vascular endothelial cells (VECs) is also regulated by linear ubiquitination. In OTULIN-KO mice, excessive linear ubiquitination of activin receptor-like kinase 1 (ALK1) by LUBAC in the bone morphogenetic protein 9 (BMP9)–ALK1–mothers against the decapentaplegic homologue 1/5 (Smad1/5) pathway leads to dysregulated proliferation and migration of VECs, ultimately resulting in angiogenic defects and embryonic lethality. These defects can be rescued either by BMP9 stimulation or through ALK1 activation [41].

**Fig. 2.**
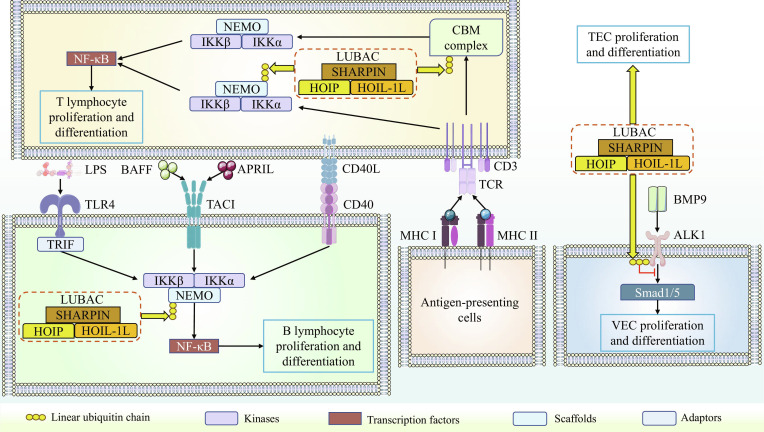
Linear ubiquitination in the regulation of key steps governing cell proliferation and differentiation. Membrane receptors such as TLR4, TACI, and CD40 regulate B-cell proliferation and differentiation by promoting NF-κB activation. The activity of the IKK complex, which is required for NF-κB activation in these pathways, is controlled by LUBAC. T-cell proliferation and differentiation are governed by the TCR–NF-κB pathway. When the TCR receives external signals, such as antigen information presented by antigen-presenting cells, it can activate IKK through the linearly ubiquitinated CBM complex. In this process, NEMO activation occurs without the need for its linear ubiquitination, leading to subsequent NF-κB activation. Alternatively, the TCR can also promote NEMO activation in a LUBAC-dependent manner, thereby driving downstream NF-κB activity. In addition, the BMP9-ALK1-Smad1/5 pathway regulates the development of VECs. Excessive linear ubiquitination of ALK1 by LUBAC impairs the proliferation and migration of these cells. Meanwhile, LUBAC is also involved in modulating the development of TECs.

As key components of the adaptive immune response, the proliferation and differentiation of both B lymphocytes and T lymphocytes are regulated by LUBAC, indicating that LUBAC is a critical regulatory molecule in the body’s defense against pathogen invasion. It is noteworthy that the BCR–NF-κB pathway in mice is not affected by LUBAC deficiency. We propose that this may represent a compensatory mechanism in B cells, helping them counteract certain developmental inhibition or death threats. Interestingly, NEMO can be activated in a LUBAC-independent manner in Jurkat cells with the assistance of the linearly ubiquitinated CBM complex. This finding is particularly intriguing as it challenges the conventional view that NEMO activation strictly depends on linear ubiquitination. This may provide an explanation for certain research observations where impaired linear ubiquitination of NEMO coexists with normal NF-κB activation. Research findings on mouse VECs remind us that excessive linear ubiquitination can exert toxic effects on cell proliferation and differentiation. Although this has not been confirmed in immune cells, it should be taken into consideration in all studies related to cell proliferation and differentiation. In summary, LUBAC activity serves as the driving force for cell (particularly immune cell) proliferation and differentiation, but precise regulation of its activity is crucial for maintaining cellular homeostasis. Figure 2 outlines how linear ubiquitination regulates cell proliferation and differentiation.

## Linear Ubiquitination: Involvement in Cell Senescence

### Overview of cell senescence

Cell senescence is a state in which replication-competent cells enter persistent cell cycle arrest and undergo diverse phenotypic changes in response to stress or injury. It serves as an important biological process that limits tissue damage, suppresses tumorigenesis, and facilitates embryonic development [42]. However, excessive accumulation of senescent cells impairs tissue regenerative capacity, induces tissue dysfunction, and can promote tumor development in a non-cell-autonomous manner [42]. Senescent cells have a well-recognized set of core features, which include 9 major senescence hallmarks: (a) cell cycle arrest; p53–p21–retinoblastoma protein (RB) and p16^INK4A^ (inhibitor of CDK4)–RB are 2 major signaling pathways that initiate and maintain cell cycle arrest; (b) the senescence-associated secretory phenotype (SASP); SASP is mainly characterized by the activation and release of a series of inflammatory factors, growth factors, proteases, and other biologically active substances; (c) chromatin alterations; PTMs of histones and DNA methylation lead to overall loss and localized gain of heterochromatin during senescence; (d) activation of DNA damage response (DDR); DDR triggers cell senescence through ataxia-telangiectasia mutated (ATM)- and ataxia-telangiectasia and Rad3 related (ATR)-mediated stabilization of p53, which activates p21 to impede cell cycle progression; (e) up-regulation of anti-apoptotic pathways; (f) increased lysosomal content; (g) metabolic adaptation; (h) altered cell surface marker proteins; and (i) altered cell morphology [43,44]. Nevertheless, no single marker is sufficient to define senescence due to the potential overlap of these features with other cell states; thus, identifying senescent cells requires a combination of multiple markers.

### Linear ubiquitination and cell senescence

Nuclear DNA damage caused by various factors is a central trigger for cell senescence. This damage, primarily in the form of DNA double-strand breaks (DSBs), leads to subsequent activation of the DDR pathway. LUBAC plays critical roles in regulating key steps of the DDR pathway. Specifically, DSBs induce autophosphorylation of ATM and ATR. Upon activation, ATM phosphorylates checkpoint kinase-2 (CHK2) while ATR phosphorylates CHK1, triggering the activation signal of the p53–p21 pathway [42,45]. p21 blocks the activity of various cyclin–CDK complexes, resulting in the formation of a complex between hypophosphorylated RB and the E2F transcription factor (E2F)/dimerization partner heterodimer. This complex then binds to the promoters of target genes to repress transcription of cell cycle-related genes [46]. In addition, activated ATM promotes phosphorylation and monoubiquitination of NEMO, both of which subsequently form a complex and are exported to the cytoplasm to assemble into the ATM–IKK complex, which ultimately drives the activation of NF-κB and subsequent expression of SASP components [42,47]. In both human and mouse cells, ATM promotes the nuclear export of NEMO, enabling its effective linear ubiquitination by LUBAC in the cytoplasm. This modification is a crucial prerequisite for the proper activation of NF-κB, which subsequently leads to the suppression of DNA damage-induced apoptosis. This phenomenon aligns with the characteristic increased resistance to apoptosis observed in senescent cells [47]. Scholars have further underscored the crucial role of linear ubiquitination in DNA damage-induced NF-κB activation. They emphasize that the ATM/NEMO-dependent NF-κB activation following DNA damage is directly linked to the induction of SASP [48].

LUBAC also orchestrates the repair mechanisms following DNA damage. DSBs can be repaired through 2 main pathways: non-homologous end joining and homologous recombination (HR). When the repair process against DSBs is inhibited or the repair process is unable to withstand the persistent DNA damage, DDR signaling will be prolonged, which ultimately leads to cell senescence. DDR pathway activated by DSBs has been pointed out to be one of the major pathways regulated by ubiquitination in cells [49]. RAD18 E3 ubiquitin protein ligase (RAD18) is a key enzyme involved in DNA damage repair. In HEK-293T cells, RAD18 is recruited to Met1- and K63-linked ubiquitin chains on chromatin proteins at DSB sites [49]. Direct binding of RAD18 to these ubiquitin chains promotes HR-mediated DSB repair. Importantly, the ubiquitination of chromatin proteins is essential for efficient recruitment and proper retention of RAD18 at DSB sites [49,50]. Additionally, ring finger protein 8 (RNF8) is another important E3 ubiquitin ligase that promotes DSB repair. Tax, the human T-cell leukemia virus type 1 (HTLV-1) viral oncoprotein, hijacks RNF8 into the cytoplasm to induce assembly of K63-linked ubiquitin chains, thereby promoting activation of the transforming growth factor β-activated kinase 1 (TAK1) complex (composed of TAK1, TAK1-binding protein 1 [TAB1], and TAB2/3) in human cells [51]. The K63-linked ubiquitin chains then recruit LUBAC and utilize it to generate the K63/Met1-linked hybrid ubiquitin chains, resulting in the activation of the IKK complex (composed of NEMO, IKKα, and IKKβ) as well as deregulation of NF-κB inhibition [51]. It has been established that over-activation of NF-κB by Tax leads to substantial accumulation of p21 and p27 proteins, thereby inducing cell cycle arrest and cell senescence. Linear ubiquitination serves as a critical molecular regulator of this process [51–53].

Intriguingly, lysosomal dysfunction has also been implicated in the onset of cell senescence. Specifically, impaired lysosomal degradation has been clearly identified as a characteristic feature of senescent cells [54]. Bleomycin treatment induces DNA damage and reactive oxygen species (ROS) generation in HT22 cells. The resulting ROS triggers lysosomal membrane permeabilization (LMP) and impairs lysosomal degradative capacity, thereby obstructing autophagic flux and ultimately leading to cell senescence [54]. In human glioblastoma (GBM) cells, LUBAC deposits Met1-linked ubiquitin chains onto damaged lysosomes that have undergone LMP, as a mechanism to restore lysosomal autophagic degradation and lysosomal homeostasis [55]. Therefore, linear ubiquitination is also potentially linked to cell senescence induced by lysosomal damage.

**Fig. 3.**
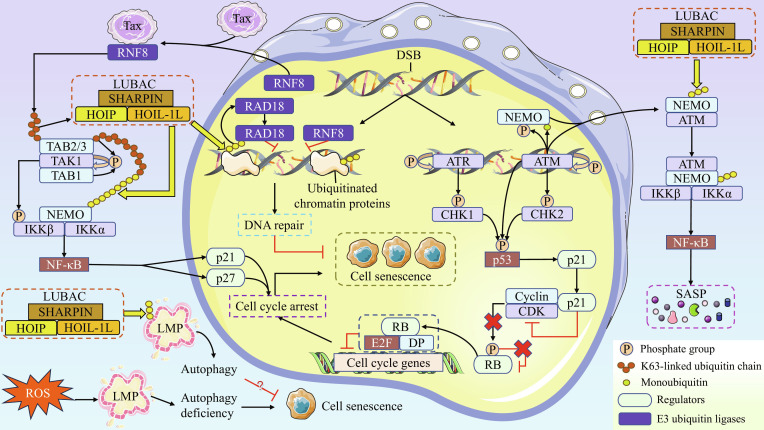
Linear ubiquitination regulates cell senescence through multiple pathways. The presence of DSBs drives the activation of ATR and ATM, which subsequently initiate the p53–p21–RB pathway, ultimately resulting in cell cycle arrest and cell senescence. Meanwhile, ATM promotes the monoubiquitination and phosphorylation of NEMO. The modified NEMO is then exported to the cytoplasm in complex with ATM, where they assemble into the ATM–IKK complex, a process in which ATM critically contributes to the linear ubiquitination of NEMO. Upon activation, the ATM–IKK complex induces SASP through NF-κB activation. DSB formation also initiates a self-directed repair response. LUBAC assembles linear ubiquitin chains on chromatin proteins at DSB sites, which then recruit and bind RAD18 to promote DSB repair. Separately, the DNA repair protein RNF8 is hijacked into the cytoplasm by the viral Tax protein, where it induces assembly of K63-linked ubiquitin chains on the TAB2/3 components of the TAK1 complex. These K63-linked chains subsequently recruit LUBAC, prompting the formation of K63/Met1-linked hybrid ubiquitin chains that are recognized and bound by NEMO. The activated IKK complex then stimulates NF-κB, contributing to substantial accumulation of p21 and p27 proteins and thereby inducing cell senescence. In addition, bleomycin-induced ROS production triggers LMP and impairs lysosomal degradation, which together block autophagic flux and promote cell senescence. The accumulation of linear ubiquitin chains on damaged lysosomes restores autophagic degradation and reestablishes lysosomal homeostasis, which may subsequently suppress cell senescence.

LUBAC serves not only as a key responder to cell senescence but also as an indispensable factor in counteracting it, highlighting its comprehensive role in regulating this process. Current evidence indicates that LUBAC-mediated senescence regulation primarily operates in the context of 3 major biological processes: DDR, pathogen invasion, and lysosomal impairment. We propose that in response to DNA damage, LUBAC orchestrates the SASP to maintain the senescent state and inhibit cell proliferation, while concurrently facilitating DSB repair. Subsequently, upon repair completion, LUBAC contributes to senescence termination. We hypothesize that this dynamic mechanism enables LUBAC to suppress disease development, such as cancer, and maintain organismal homeostasis. The mechanism by which HTLV-1 exploits LUBAC to induce senescence is particularly intriguing. Given that senescent cells exhibit high resistance to apoptosis and long-term persistence, we speculate that HTLV-1 leverages cell senescence as a strategy to establish a stable and persistent latent environment, thereby evading immune surveillance. This hypothesis aligns with the clinical observation that HTLV-1 can remain latent in the human body for decades, even for life. Furthermore, although studies exploring LUBAC in lysosomal damage and those linking lysosomal damage to senescence have utilized different cell lines, these models are all neural-derived. Notably, it has been demonstrated that damaged lysosomes in both human and mouse normal neurons are modified by linear ubiquitin chains [55]. Therefore, we consider investigating the relationship between LUBAC and cell senescence from the perspective of lysosomal damage to be a highly meaningful direction for future research. Figure 3 vividly demonstrates the strong link between linear ubiquitination and cell senescence.

## Linear Ubiquitination: Regulation of Cell Survival/Death

The maintenance of the equilibrium between cell survival and death is a prerequisite for the normal progress of physiological activities in the body, and linear ubiquitination, as a regulatory pivot, is a key point to maintaining the balance of cell survival and death. Therefore, an in-depth study of the mechanisms of linear ubiquitination in the regulation of cell survival and death will greatly enhance our understanding of cell fate determination and related diseases. Since cell survival and death are opposites of each other, this section mainly focuses on the various patterns of cell death as an entry point to explore the key regulatory role of linear ubiquitination in cell survival and death decisions.

### Linear ubiquitination and apoptosis

#### Overview of apoptosis

Apoptosis, a genetically regulated form of programmed cell death, is mainly initiated via 2 separate mechanisms: the intrinsic and extrinsic pathways. In the intrinsic pathway, stimuli such as DNA damage promote the alteration of outer mitochondrial membrane (OMM) permeability by B-cell lymphoma 2 (BCL2)-associated X (BAX) and BCL2 antagonist killer (BAK). This leads to the release of cytochrome c (Cyt c) from the mitochondrial intermembrane space into the cytosol, thereby activating the executioner caspase-3/7 and ultimately initiating intrinsic apoptosis [56]. The entire process is also subject to regulation by anti-apoptotic molecules (e.g., BCL2, BCL-extra large [BCL-XL], and myeloid cell leukemia 1 [MCL1]), which, through their interaction with pro-apoptotic proteins such as BAX and BAK, inhibit Cyt c release, thereby executing their anti-apoptotic function [57].

When various factors induce death ligands (e.g., Fas ligand [FasL], TNF-related apoptosis-inducing ligand [TRAIL], and TNF-α) to bind to their respective membrane receptors (e.g., Fas, death receptor 4/5 [DR4/5], and TNF receptor 1 [TNFR1]), Fas-associated protein with death domain (FADD) or TNFR1-associated death domain protein (TRADD) is recruited to the corresponding membrane receptor, and the different membrane receptors then go on to recruit distinct key proteins to generate receptor-associated complexes [58]. These complexes recruit procaspase-8, with the subsequent death signaling promoting its cleavage and activation, ultimately leading to the activation of caspase-3/7 and the initiation of extrinsic apoptosis [58].

#### Linear ubiquitination and apoptosis

Current research on the relationship between linear ubiquitination and intrinsic apoptosis remains limited. In the retinoic acid-inducible gene I (RIG-I)-induced apoptotic pathway, interferon regulatory factor 3 (IRF3) binds to BAX and facilitates its translocation to mitochondria, leading to subsequent Cyt c release and apoptosis in HT1080 cells. This process proceeds efficiently only when IRF3 undergoes linear ubiquitination, and it is effectively suppressed by OTULIN [59]. Furthermore, in human colorectal cancer (CRC) cells, matrine treatment markedly down-regulates SHARPIN and BCL2 expression while up-regulating caspase-3, caspase-8, and BAX levels, collectively inducing apoptosis [60]. In mice, loss of SHARPIN not only causes prominent epidermal hyperplasia but also promotes substantial intrinsic apoptosis in keratinocytes [61]. Notably, SHARPIN and the LUBAC complex exhibit discordant regulatory functions in intrinsic apoptosis. Although both are generally considered pro-survival factors, LUBAC can paradoxically promote apoptosis in specific contexts, such as the RIG-I-mediated pathway. We therefore hypothesize that the nature of the pro-death signal is a key determinant underlying these context-dependent regulatory outcomes.

Currently, LUBAC-mediated linear ubiquitination has been shown to be more involved in regulating TNF-α-induced extrinsic apoptosis compared to intrinsic apoptosis. In general, TNF-α signaling activation does not typically induce cell death in mice and humans. Cells only initiate the TNF-α-mediated cell death pathway under severe cellular stress [62]. The binding of TNF-α to TNFR1 promotes rapid assembly of intracellular complex I, containing proteins such as receptor-interacting protein kinase 1 (RIPK1), TNFR1, TRADD, LUBAC, CYLD, and SPATA2 [63]. RIPK1, TNFR1, and TRADD are modified by K63- and Met1-linked ubiquitin chains. The K63/Met1-linked hybrid ubiquitin chains on RIPK1 create a platform that recruits the TAK1 complex and the IKK complex [64,65]. Subsequent activation of the IKK complex facilitates nuclear translocation of activated NF-κB, which drives expression of numerous target genes including cellular FLICE inhibitory protein (c-FLIP) [62]. c-FLIP then binds to procaspase-8 within complex II, a cytosolic complex formed after the membrane dissociation of complex I, and inhibits its activation, thereby preventing apoptosis [62]. However, c-FLIP stability is maintained only after it undergoes linear ubiquitination by LUBAC [66]. Thus, LUBAC is involved in regulating multiple key steps of the TNF-α signaling pathway and primarily acts as a pro-survival factor to suppress apoptosis.

However, disruption of linear ubiquitination homeostasis can shift TNF-α signaling from promoting survival to triggering death, thereby inducing extrinsic apoptosis. It has been demonstrated that CYLD promotes TNF-α-induced apoptosis in human cells by removing linear ubiquitin chains from complex I [65]. HOIP deficiency prevents linear ubiquitination of c-FLIP, leading to its degradation via the proteasome-dependent pathway and ultimately sensitizing HeLa cells to apoptosis [66]. Similarly, in human cells, overexpression of OTULIN eliminates linear ubiquitin chains on NEMO and promotes TNF-α-mediated apoptosis [25]. Paradoxically, knockdown of OTULIN also increases cellular susceptibility to apoptosis [25]. Other researchers have proposed a possible explanation: OTULIN releases LUBAC activity by preventing its auto-linear ubiquitination, thereby restricting TNF-α-mediated cell death in mouse models [67]. Consequently, the maintenance of linear ubiquitination homeostasis requires the participation of multiple molecules, and the linear ubiquitination status of key molecules ultimately determines whether a cell survives or undergoes death.

**Fig. 4.**
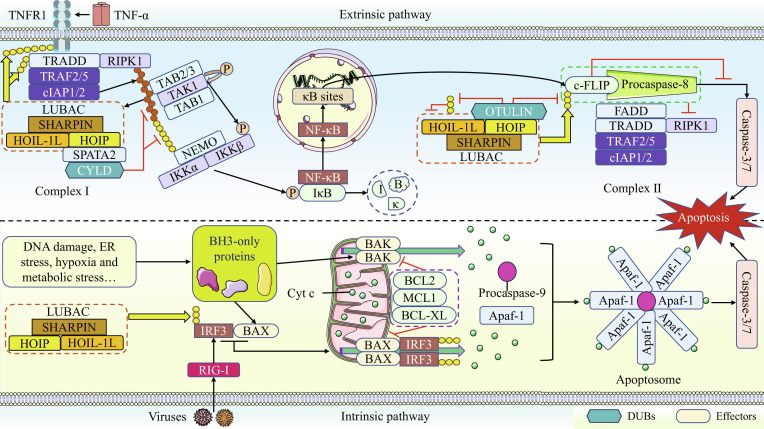
Linear ubiquitination in the regulation of key apoptosis pathway steps. During extrinsic apoptosis, TNF-α binding to TNFR1 promotes the formation of complex I, where LUBAC subsequently deposits linear ubiquitin chains on TNFR1, TRADD, and RIPK1. The K63/Met1-linked hybrid ubiquitin chains on RIPK1 recruit both the TAK1 complex and the IKK complex. Linear ubiquitination of NEMO facilitates the activation of the IKK complex, thereby inducing nuclear translocation of NF-κB and its subsequent binding to κB sites, which drives the transcription of target genes such as c-FLIP. Upon linear ubiquitination, c-FLIP stably associates with procaspase-8 in complex II and impedes its activation, ultimately suppressing apoptosis. Conversely, CYLD promotes apoptosis by removing K63- and Met1-linked ubiquitin chains from complex I. OTULIN removes linear ubiquitin chains from cytosolic proteins while relieving LUBAC autoinhibition through eliminating linear chains on LUBAC itself. Consequently, functional impairment of LUBAC leads to the execution of TNF-α-mediated extrinsic apoptosis. During intrinsic apoptosis, cellular stress signals such as DNA damage activate BH3-only proteins. These proteins subsequently facilitate the translocation of BAK and BAX to the OMM, where their oligomerization alters membrane permeability and induces Cyt c release. However, the apoptotic functions of BAK and BAX are effectively inhibited by anti-apoptotic regulators including BCL2, BCL-XL, and MCL1. When Cyt c release becomes irreversible, Cyt c assembles with procaspase-9 and apoptotic protease activating factor-1 (Apaf-1) to form the apoptosome. This complex subsequently initiates the execution of apoptosis. Within the RIG-I signaling pathway, viral RNA-activated RIG-I stimulates IRF3. Following its linear ubiquitination, IRF3 interacts with BAX and facilitates its mitochondrial translocation, thereby promoting subsequent execution of the intrinsic apoptosis program.

In summary, LUBAC exhibits a dual regulatory role in apoptosis control. Within the RIG-I-mediated apoptotic pathway, its pro-death function is hypothesized to relate to the unique stimulus of viral infection. Notably, numerous viruses exploit host cell resources for continuous replication and dissemination, while LUBAC-induced cellular collapse may prevent further viral spread to adjacent tissues. This hypothesis is further supported by the observation that RIG-I-mediated apoptosis predominantly occurs during later stages of viral infection [59]. The pro-survival function demonstrated by SHARPIN appears to operate through LUBAC-dependent mechanisms. Furthermore, LUBAC acts as a definitive negative regulator in TNF-α-mediated extrinsic apoptosis, where its ubiquitination modifications on multiple proteins collectively confer resistance to cell death. Nevertheless, we note that OTULIN knockdown also triggers cell death due to ensuing excessive auto-linear ubiquitination of LUBAC. Consequently, properly regulated linear ubiquitination is essential for maintaining cellular homeostasis. Figure 4 clearly illustrates the key steps through which LUBAC regulates apoptosis.

### Linear ubiquitination and necroptosis

#### Overview of necroptosis

Necroptosis is a programmed and regulated form of necrotic cell death, primarily mediated by specific ligand/receptor pairs such as TNF-α/TNFR1; FasL/Fas; double-stranded RNA/TLR3; LPS/TLR4; and type I interferons (IFNs)/IFN α/β receptor (IFNAR) [68]. For instance, the pathway mediated by membrane receptors such as TNFR1 and Fas generates complexes containing proteins like FADD, RIPK1, and RIPK3 upon death signal reception. When caspase-8 is deleted or inhibited, necrosomes are then generated, leading to mixed lineage kinase domain-like protein (MLKL) activation, which disrupts plasma membrane integrity and induces necroptosis [58]. TLR3/4 can recruit and activate RIPK1 and RIPK3 through the TIR domain-containing adaptor inducing IFN-β, thereby inducing necroptosis [58]. Furthermore, TLR3/4 can also activate RIPK3 and initiate necroptosis independently of RIPK1 [68]. During the continuous replication of viruses like influenza A virus and murine cytomegalovirus, the production of z-RNA/DNA activates Z-DNA binding protein 1 (ZBP1) within the infected cell. ZBP1 then directly recruits RIPK3 to initiate necroptosis, bypassing RIPK1 entirely [68]. IFNs not only promote necrosome assembly via the Janus kinase (JAK)/signal transduction and activator of transcription (STAT) signaling pathway to induce necroptosis, but also trigger ZBP1–RIPK3–MLKL complex formation, executing cell death program [68]. The above signaling pathways constitute the regulatory network of necroptosis.

#### Linear ubiquitination and necroptosis

To date, the majority of research on the regulation of necroptosis through linear ubiquitination has centered on the TNF-α/TNFR1 pathway, which is regarded as the primary executor of necroptosis. Specifically, when RIPK1 is deubiquitinated by CYLD, complex I dissociates from the membrane and associates with FADD, procaspase-8, and c-FLIP to form the stable complex IIa [69,70]. Conversely, when the failure of RIPK1 ubiquitination occurs due to the depletion of cellular inhibitor of apoptosis protein-1/2 (cIAP1/2) or the inhibition of TAK1 and/or LUBAC, the non-ubiquitinated RIPK1 translocates to the cytosol, where it forms complex IIb with RIPK3, FADD, c-FLIP, and procaspase-8. Under conditions where caspase-8 is absent or inhibited, RIPK1 within both complex IIa and IIb interacts with RIPK3 and MLKL to form the necrosome, culminating in the induction of necroptosis [69,70]. Therefore, LUBAC serves as a critical inhibitory molecule of necroptosis. Both the removal of linear ubiquitin chains by CYLD and the dysfunction of LUBAC itself create the necessary conditions for the initiation of necroptosis.

Accumulating evidence has further substantiated the inhibitory role of LUBAC in necroptosis, with most studies focusing on the kinase activity status of RIPK1. It has been demonstrated that deficiency in cIAP1/2 or LUBAC sensitizes cells to TNF-α-induced necroptosis, which depends on the kinase activity of RIPK1 [71]. In mice, the K612 residue of RIPK1 serves as a major site for linear ubiquitination, and linear ubiquitination at this site effectively suppresses both TNF-α-mediated necroptosis and apoptosis, thereby further restraining the development of systemic inflammation [72]. Moreover, LUBAC deficiency in complex I causes impaired recruitment of A20-binding inhibitor of NF-κB-1 and A20, leading to increased K63 ubiquitination and enhanced activity of RIPK1 within complex I, which ultimately triggers necroptosis in mouse embryonic fibroblasts (MEFs) [73]. These findings reveal an intriguing fact: K63 ubiquitination of RIPK1 in complex I does not solely promote cell survival; instead, uncontrolled K63 ubiquitination of RIPK1 can drive cells toward necroptosis. Beyond complex I, RIPK1 within the necrosome is also modified by linear ubiquitination. Surprisingly, in HT29 cells pretreated with IAP antagonists, the linear ubiquitination status of RIPK1 in the necrosome neither affects necrosome assembly nor influences the progression of necroptosis [74]. Therefore, based on current evidence, it is highly likely that the functional consequences of RIPK1 linear ubiquitination in complex I differ from those in the necrosome with regard to the regulation of necroptosis. In addition, DUBs that target linear ubiquitin chains also modulate necroptosis. For instance, knockdown of either SPATA2 or CYLD protects L929 cells from necroptosis [75]. Further studies indicate that SPATA2 deficiency suppresses RIPK1 kinase activity by promoting its linear ubiquitination, thereby conferring resistance to both apoptosis and necroptosis in MEFs [29]. Thus, when direct modulation of LUBAC activity is challenging, targeting DUBs may represent an alternative strategy for regulating linear ubiquitination-mediated necroptosis.

However, a considerable body of research challenges the role of LUBAC as a negative regulator of necroptosis. In HT29 cells, LUBAC activity has been shown to be required for TNF-α-mediated necroptosis. Rather than interfering with the necroptotic phosphorylation of RIPK1, RIPK3, and MLKL, necrosome formation, or MLKL oligomerization, LUBAC drives necroptosis by promoting the membrane accumulation of MLKL [76]. Furthermore, during necroptosis in HT29 cells, the levels of both K63 and linear ubiquitination of RIPK1 are markedly enhanced. Notably, the K115R mutation in RIPK1 not only markedly abolishes this enhancement but also disrupts necrosome assembly and suppresses necroptosis [77]. The authors demonstrated that the necroptotic ubiquitination of RIPK1 is essential for maintaining its kinase activity within the necrosome. On the other hand, depletion of OTULIN not only promotes necroptosis in HaCaT cells but also enhances linear ubiquitination of RIPK1 and inactivates CYLD [78]. The authors propose that increased linear ubiquitination is associated with heightened sensitivity to cell death. However, we posit that this enhancement may merely be a consequence of OTULIN deficiency, rather than a direct driver of necroptosis. For instance, OTULIN loss inactivates CYLD, thereby impairing its antagonism of LUBAC and ultimately promoting linear ubiquitination.

**Fig. 5.**
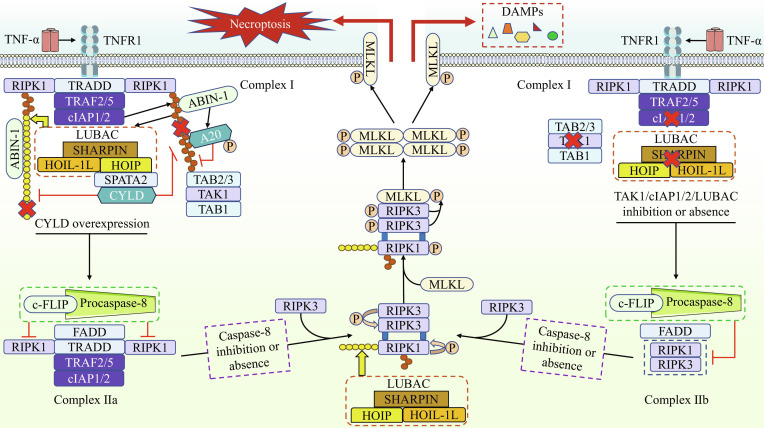
Linear ubiquitination participates in the regulation of the necroptosis pathway. Stimulation of TNFR1 by TNF-α induces the formation of complex I. When proteins within complex I are properly modified by K63- and Met1-linked ubiquitin chains, the downstream NF-κB-mediated pro-survival signaling pathway is normally activated. CYLD promotes the formation of complex IIa by removing K63- and/or Met1-linked ubiquitin chains from RIPK1. In addition to the key molecules present in complex I, complex IIa also incorporates FADD, procaspase-8, and c-FLIP. When cIAP1/2 are depleted or TAK1 and/or LUBAC are inhibited, non-ubiquitinated RIPK1 translocates to the cytoplasm and forms a TRADD-independent complex IIb with RIPK3, FADD, c-FLIP, and procaspase-8. Under conditions of caspase-8 absence or inhibition, RIPK3 is recruited to RIPK1. RIPK1 activates RIPK3 through cis-autophosphorylation, after which RIPK3 promotes autophosphorylation via homodimerization. Activated RIPK3 further recruits MLKL and facilitates its phosphorylation. Subsequently, MLKL forms oligomers and translocates to the plasma membrane, leading to membrane disruption and ultimately resulting in necroptosis and the release of damage-associated molecular patterns (DAMPs). LUBAC exerts its influence on the necroptotic process mainly by modifying RIPK1. Nevertheless, the functional significance of Met1-linked ubiquitin chains on RIPK1 in the necrosome has not yet been clarified.

The role of LUBAC activity in regulating necroptosis appears to be context-dependent. It has been observed that the anti-necroptotic function of LUBAC is more clearly defined in mice, whereas in human cells, LUBAC seems to predominantly promote necroptosis. We propose that the regulatory effect of LUBAC on necroptosis may involve underlying cell type-specific and species-dependent mechanisms. Furthermore, even within the same HT-29 cell model, the conclusion that linear ubiquitination contributes to necroptosis remains contentious among researchers, which may be attributed to the use of IAP antagonists by some groups [74]. Notably, different studies have presented conflicting views regarding whether linear ubiquitination promotes RIPK1 kinase activity within the necrosome in HT-29 cells [76,77]. Based on comprehensive analysis, we suggest that K63 ubiquitination may be the primary contributor to maintaining RIPK1 kinase activity in the necrosome of HT-29 cells. Additionally, the functional consequences of RIPK1 linear ubiquitination may differ across molecular complexes (e.g., complex I versus the necrosome), and thus, these contexts should be examined separately in relevant studies. In summary, the precise role of linear ubiquitination in necroptosis and its regulatory mechanisms require further elucidation. The modification and regulation of important nodes in necroptosis by linear ubiquitination is then demonstrated in Fig. 5.

### Linear ubiquitination and autophagy-dependent cell death

#### Overview of autophagy-dependent cell death

Autophagy is a conserved catabolic process, primarily classified into 3 types: macroautophagy, microautophagy, and chaperone-mediated autophagy (CMA). Under most physiological conditions, autophagy serves a cytoprotective role and negatively regulates cell death [79]. Under specific circumstances, autophagy also serves as a key mechanism responsible for cell death. It is now established that autophagy regulates virtually all forms of cell death in disease states [80]. The key mechanisms for autophagy-induced cell death include both the unrestricted degradation of cellular components from excessive autophagy and the accumulation of autophagosomes due to compromised autophagic flux [80,81]. Based on established guidelines, the field delineates 3 forms of cell death pertinent to autophagy: (a) autophagy-associated cell death; (b) autophagy-mediated cell death; and (c) autophagy-dependent cell death [82].

Autophagy-dependent cell death is a form of programmed cell death that strictly relies on the autophagy machinery (or its components) and operates independently of other cell death modalities [80]. Currently, the molecular pathways driving autophagy-dependent cell death remain incompletely elucidated. However, the following 4 criteria serve as mandatory conditions for defining autophagy-dependent cell death: (a) autophagic flux must be elevated during cell death; (b) the death process must be reversible through genetic or pharmacological inhibition of autophagy; (c) cell death must depend on at least 2 distinct autophagy-related molecules; and (d) the process must occur in the absence of other cell death modalities [80].

#### Linear ubiquitination and autophagy-dependent cell death

The initial step in triggering autophagy-dependent cell death pathways is the induction of autophagy, with mitophagy being the most extensively studied form of autophagy to date. Mitophagy refers to the selective degradation of damaged mitochondria under conditions of mitochondrial stress, playing a crucial role in maintaining mitochondrial quality control and cellular homeostasis [83]. Excessive activation of mitophagy has been proven to induce cell death, which may be attributed to the over-degradation of functional mitochondria and subsequent disruption of mitochondrial homeostasis [84]. Linear ubiquitin chains on mitochondria are essential for efficient Parkin-mediated mitophagy in HeLa cells [85]. Specifically, phosphatase and tensin homolog-induced putative kinase 1 (PINK1) on mitochondria first phosphorylates linear ubiquitin chains. The phosphorylated ubiquitin chains then recruit phosphorylated Parkin to the mitochondria and interact with it, thereby promoting the amplification of local phosphorylated-ubiquitin signal and the subsequent recruitment of autophagy receptors such as optineurin (OPTN). This cascade ultimately facilitates the autophagic clearance of damaged mitochondria [85,86]. In human GBM cells, the compound F0911-7667 induces mitophagy-dependent cell death via the sirtuin-1–PINK1–Parkin pathway [87]. Additionally, cannabidiol has been shown to induce cell death in human GBM cells by triggering endoplasmic reticulum stress to activate PINK1/Parkin-mediated lethal mitophagy [88]. Given the pivotal role of linear ubiquitin chains in Parkin-mediated mitophagy, it is reasonable to conclude that LUBAC activity is crucial for mitophagy-mediated cell death.

Distinct lysophagy also serves as a key process regulating autophagy-dependent cell death. LMP or complete lysosomal rupture represents a common and severe stress condition. When repair mechanisms fail, these damaged lysosomes are tagged with ubiquitin and eliminated via the selective macroautophagy pathway, a process known as lysophagy. Studies have demonstrated that loperamide and pimozide disrupt lipid homeostasis and induce excessive autophagy in human GBM cells through synergistic effects. This process induces severe autophagy-dependent LMP, consequently leading to cell death through a mechanism the authors have specifically termed “autophagy-dependent lysosomal cell death”. [89] Interestingly, lysophagy, the selective autophagy of damaged lysosomes, promotes GBM cell survival in this context [89]. This phenomenon is largely attributed to linear ubiquitination. Specifically, lysosomes that have undergone LMP are decorated with linear ubiquitin chains, a process dependent on pre-existing K63-linked ubiquitin chains. This linear ubiquitination, in turn, facilitates human GBM cell survival by driving lysophagy [55]. Furthermore, linear ubiquitin chains are also deposited on damaged lysosomes in normal neuronal cells of both mice and humans [55]. In summary, these findings demonstrate that in human neurons, LUBAC-mediated linear ubiquitination facilitates the clearance of damaged lysosomes through lysophagy, thereby establishing a protective mechanism against autophagy-dependent lysosomal cell death.

**Fig. 6.**
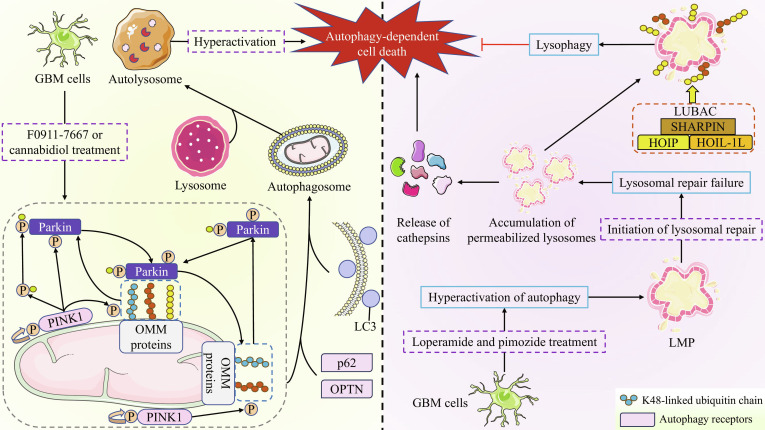
Mechanism by which linear ubiquitination regulates autophagy-dependent cell death. In GBM cells, treatment with compound F0911-7667 or cannabidiol induces stabilization of PINK1 on the OMM. Subsequently, PINK1 activates Parkin and phosphorylates pre-existing ubiquitin chains (K48-, K63-, and potentially Met1-linked) on mitochondria. The phosphorylated ubiquitin chains recruit activated Parkin, promoting further ubiquitination of various OMM proteins. Persistent activation of this process drives mitochondrial degradation via mitophagy, and excessive mitophagy ultimately leads to autophagy-dependent cell death. Alternatively, treatment with loperamide and pimozide causes hyperactivation of autophagy, resulting in severe autophagy-dependent LMP. The lysosomal repair machinery fails to restore these damaged lysosomes, leading to accumulation of permeabilized lysosomes and subsequent release of lysosomal contents (e.g., cathepsins), which eventually triggers autophagy-dependent cell death. On the other hand, the LUBAC deposits linear ubiquitin chains on damaged lysosomes in a K63 ubiquitination-dependent manner, thereby targeting them for clearance via lysophagy to promote cell survival.

It is evident that the regulation of autophagy-dependent cell death by linear ubiquitination is primarily observed in cancer cells, particularly in GBM. In mitophagy-dependent cell death, LUBAC acts as a potential facilitator. This suggests that promoting LUBAC-mediated linear ubiquitination to activate this pathway may represent a highly promising interventional strategy for cancer therapy. Conversely, in autophagy-dependent lysosomal cell death, LUBAC suppresses the execution of the cell death program via lysophagy. In our view, the suppressive role of LUBAC in autophagy-dependent cell death has an adverse clinical implication for GBM treatment, by potentially inducing resistance to specific therapeutic drugs in patients. Collectively, the divergent roles of LUBAC in autophagy-dependent cell death highlight the complexity of linear ubiquitination-mediated regulation in tumors. Further studies are needed to elucidate the potential association between linear ubiquitination, autophagy-dependent cell death, and cancer cells. Figure 6 illustrates the key mechanistic steps involving linear ubiquitination in regulating autophagy-dependent cell death.

### Linear ubiquitination and pyroptosis

#### Overview of pyroptosis

Pyroptosis is a form of programmed cell death intimately linked to inflammation. To date, 4 distinct pathways triggering pyroptosis have been identified: the canonical inflammasome pathway, the noncanonical inflammasome pathway, the caspase-3-mediated pathway, and the granzyme-mediated pathway. Overall, pyroptosis occurs predominantly in an inflammasome-dependent manner [90]. In the canonical inflammasome pathway, pattern recognition receptors (PRRs), such as nucleotide-binding oligomerization domain (NOD)-like receptor family pyrin domain containing 3 (NLRP3), recognize pathogen-associated molecular patterns (PAMPs) or damage-associated molecular patterns (DAMPs). This recognition prompts the PRR to recruit the adapter protein apoptosis-associated speck-like protein containing a CARD (ASC) and procaspase-1, assembling the inflammasome complex. Subsequently, procaspase-1 undergoes autocleavage into its active form, which then cleaves the inactive precursors of interleukin-1β (IL-1β) and IL-18, as well as gasdermin D (GSDMD). The N-terminal fragment of GSDMD (GSDMD-N) subsequently oligomerizes and forms pores in the plasma membrane, ultimately triggering the inflammatory response and pyroptosis. The noncanonical inflammasome pathway is triggered by the direct engagement of LPS with human caspases-4/5 or murine caspase-11. The activation of these caspases leads to the cleavage of GSDMD, thereby initiating pyroptosis. Concurrently, the GSDMD-N pores can activate the NLRP3 and caspase-1, leading to the maturation and release of IL-18 and IL-1β [91].

#### Linear ubiquitination and pyroptosis

The relationship between LUBAC and pyroptosis is intricate and complex, with its activity appearing to promote the progression of pyroptosis. LUBAC has been demonstrated to be essential for NLRP3 inflammasome activation in bone marrow-derived macrophages (BMDMs) [92]. Specifically, LUBAC induces inflammasome activation by facilitating the assembly of the NLRP3/ASC inflammasome, which subsequently promotes the cleavage and activation of procaspase-1 and the secretion of IL-1β, a process independent of NF-κB activation [92]. Furthermore, the authors showed that ASC in both BMDMs and 293T cells is a substrate of LUBAC, undergoing pronounced linear ubiquitination upon NLRP3 stimulation [92]. However, the specific impact of ASC linear ubiquitination on NLRP3 inflammasome-mediated pyroptosis remains unclear. SHARPIN has been identified as required for NLRP3 inflammasome activation via both canonical and noncanonical pathways in BMDMs, though it is not involved in the activation of the NLR family CARD domain-containing protein 4 or absent in melanoma 2 inflammasomes [93]. Moreover, the absence of SHARPIN results in a failure to activate caspase-1 and consequently inhibits the production of IL-1β and IL-18 [93]. It is evident that the NLRP3 inflammasome in BMDMs is a primary target of LUBAC intervention. Both LUBAC and its subunit SHARPIN potentially provide the driving force for pyroptosis through their role in inflammasome activation.

Notably, the inhibitory role of LUBAC in pyroptosis also appears to be supported by multiple lines of evidence. In a rat model of osteoarthritis (OA), linear ubiquitination of liver kinase B1 (LKB1) enhances its activity, leading to AMP-activated protein kinase (AMPK) pathway activation and subsequent suppression of the NLRP3 inflammasome response, thereby inhibiting chondrocyte pyroptosis [94]. In HaCaT cells, LUBAC can physically interact with procaspase-1 and mediate its linear ubiquitination, resulting in the suppression of caspase-1 function. Depletion of either HOIP or SHARPIN leads to enhanced caspase-1 activation and promotes cell death upon inflammasome stimulation [95]. Furthermore, A20, a ubiquitin-editing enzyme, appears to have a unique connection with linear ubiquitination and pyroptosis. Studies in MEFs and HaCaT cells have confirmed that A20 directly binds to linear ubiquitin chains via its ZF7 domain, thereby stabilizing them, and this function contributes to protection against cell death [96]. Importantly, A20-knockout mice exhibit enhanced NLRP3 inflammasome activation, increased IL-1β secretion, and develop arthritis and cartilage destruction [97]. Collectively, these findings reveal a potential relationship between A20 and pyroptosis: A20 seems to function as a negative regulator of pyroptosis, likely through its linear ubiquitin-stabilizing activity.

**Fig. 7.**
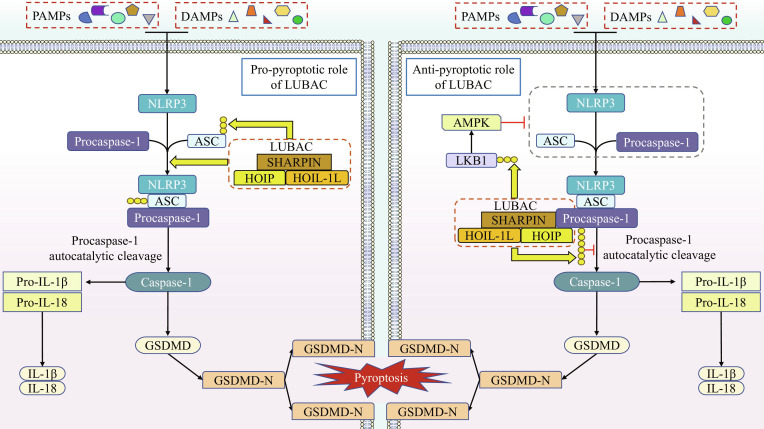
Linear ubiquitination regulates pyroptosis through dual mechanisms. Pro-pyroptotic role of LUBAC: Upon intracellular signaling triggered by PAMPs or DAMPs, NLRP3, procaspase-1, and ASC assemble into the inflammasome complex, wherein ASC undergoes linear ubiquitination while LUBAC provides the impetus for inflammasome assembly. Subsequently, procaspase-1 is cleaved and activated into caspase-1, which facilitates the translocation of GSDMD-N to the plasma membrane. This leads to pore formation, triggering pyroptosis and concomitant release of IL-1β/IL-18. Anti-pyroptotic role of LUBAC: Following inflammasome assembly stimulated by PAMPs/DAMPs, LUBAC interacts with and mediates linear ubiquitination of procaspase-1, thereby preventing its activation. Furthermore, LUBAC catalyzes linear ubiquitination on LKB1 to enhance its activity, driving subsequent AMPK activation that suppresses expression of inflammasome-related proteins. Ultimately, through these coordinated interventions, LUBAC effectively blocks the pyroptotic process.

In the aforementioned studies, the phenomenon of ASC linear ubiquitination by LUBAC is particularly intriguing, although the authors did not explicitly define its specific contribution to pyroptosis. Given the established conclusion that LUBAC induces inflammasome activation by promoting NLRP3/ASC inflammasome assembly, coupled with the fact that LUBAC stabilizes the c-FLIP/procaspase-8 complex by linearly ubiquitinating c-FLIP to prevent its degradation, we propose that Met1-linked ubiquitin chains on ASC are highly likely to positively regulate pyroptosis in BMDMs by stabilizing ASC or the NLRP3 inflammasome. Moreover, the dichotomous role of LUBAC in regulating pyroptosis provides further evidence supporting the cell-type or tissue-type specificity of its regulatory functions. The relationship between A20, pyroptosis, and linear ubiquitination remains incompletely elucidated. Nevertheless, the co-demonstration in HaCaT cells of LUBAC-mediated inflammasome suppression alongside A20’s protective stabilization of linear ubiquitin chains presents compelling evidence that cannot overlook the possibility of A20 inhibiting pyroptosis through maintaining linear ubiquitin chain stability. We contend that future scholarly investigation, whether confirming or refuting this potential mechanism, will substantially advance our mechanistic understanding of pyroptosis regulation through linear ubiquitination. The regulatory role of LUBAC in pyroptosis is visually summarized in Fig. 7.

### Linear ubiquitination and ferroptosis

#### Overview of ferroptosis

Ferroptosis is an iron-dependent form of programmed cell death driven by lethal lipid peroxidation, which results from disturbed cellular metabolism and imbalanced redox homeostasis. The generation of lipid peroxidation primarily involves 2 pathways: nonenzymatic and enzymatic [98]. Disturbances in iron excretion, impaired iron uptake, or excessive iron accumulation resulting from various factors, such as inhibition or degradation of ferroportin, accumulation of transferrin receptor (TfR) on the cell surface, or autophagic degradation of ferritin, can lead to intracellular iron overload. Under these conditions, labile Fe^2+^ reacts with hydrogen peroxide (H₂O₂), generating a large amount of hydroxyl radicals (•OH). These radicals subsequently initiate peroxidation of phospholipids (PLs) containing polyunsaturated fatty acids (PUFAs). All these processes collectively represent the nonenzymatic pathway [99,100]. In the enzymatic pathway, acyl-coenzyme A (CoA) synthetase long-chain family member 4 conjugates PUFAs to CoA to form activated fatty acyl-CoA esters. These esters are incorporated into phosphatidylethanolamine (PE) and phosphatidylcholine (PC) by lysophosphatidylcholine acyltransferase 3, yielding PL-PUFA (e.g., PE-PUFA), which subsequently undergo peroxidation catalyzed by lipoxygenases (LOXs) [101]. Furthermore, cells possess multiple defense mechanisms against ferroptosis, such as the System x_c_^–^-glutathione (GSH)–GSH peroxidase 4 (GPX4) axis and the ferroptosis suppressor protein–CoQ10 pathway. Once these protective systems are compromised, the execution phase of ferroptosis may be initiated [102].

#### Linear ubiquitination and ferroptosis

GPX4 is currently the only known mammalian enzyme capable of reducing PL hydroperoxides (PL-PUFA-OOH) to their corresponding PL alcohols (PL-PUFA-OH), while LUBAC primarily participates in the regulation of ferroptosis by modifying GPX4. In both human and murine cells, GPX4 has been identified as a key substrate of LUBAC. LUBAC targets GPX4 and catalyzes the formation of Met1-linked ubiquitin chains, thereby enhancing GPX4 stability and cellular resistance to ferroptosis. During the recruitment of LUBAC by GPX4, ubiquitination of GPX4 (such as K63 ubiquitination) may serve as a foundation for LUBAC recruitment. Moreover, the absence of HOIP leads to GPX4 degradation and accumulation of lipid peroxides, ultimately increasing cellular sensitivity to ferroptosis [103]. In the context of OA, in addition to the driving force provided by pyroptosis in its pathogenesis, ferroptosis also plays a pivotal role in the disease process. p21 enhances the linear ubiquitination of GPX4 by facilitating its recruitment to LUBAC, thereby increasing GPX4 stability. This represents a key mechanism through which murine chondrocytes resist ferroptosis. Knockdown of p21 not only promotes the accumulation of lipid peroxides induced by IL-1β and Erastin in chondrocytes but also leads to cartilage degradation in OA mice and a pronounced reduction in GPX4 levels within the cartilage [104]. Therefore, the linear ubiquitination activity of LUBAC is both essential for GPX4 function and a critical prerequisite for cellular resistance to ferroptosis.

In addition to the GPX4-mediated defense mechanism, ferritinophagy is another critical physiological process involved in the regulation of ferroptosis. Excessive ferritinophagy leads to iron overload, which, in turn, causes oxidative damage and ferroptosis. Nuclear receptor coactivator 4 (NCOA4) is a central protein regulating ferritinophagy, and it has been confirmed as a substrate of LUBAC [105]. In murine hepatocytes, OTULIN can promote the degradation of NCOA4 via the proteasomal pathway by disrupting the linear ubiquitin chains on NCOA4, thereby enhancing other types of ubiquitination of NCOA4. The reduced NCOA4 levels impair the ferritinophagy pathway, ultimately suppressing acetaminophen-induced ferroptosis [105]. This study reveals that linear ubiquitin chains stabilize NCOA4 to hinder its degradation and promote ferroptosis. Furthermore, CYLD/SPATA2 enhances ferritinophagy by removing ubiquitin chains from NCOA4, subsequently promoting doxorubicin-induced ferroptosis in murine cardiomyocytes [106]. Together, these studies provide insights into the potential link between linear ubiquitination and cellular ferroptosis from the perspectives of DUBs and NCOA4.

**Fig. 8.**
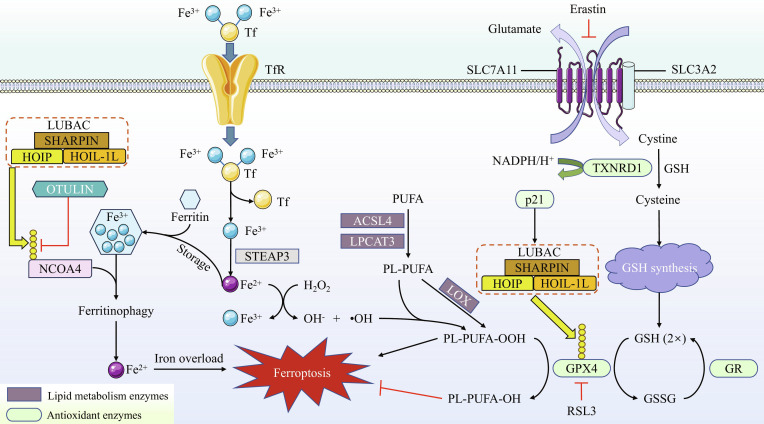
Linear ubiquitination orchestrates both the promotion and suppression pathways of ferroptosis. In the serum, Fe^3+^ binds to Tf and enters the cell via TfR on the cell membrane. Following endocytosis, Fe^3+^ is reduced to Fe^2+^ within the endosomal compartment by 6-transmembrane epithelial antigen of the prostate 3 (STEAP3). Unstable Fe^2+^ reacts with H_2_O_2_ to generate •OH. The overproduction of •OH drives widespread peroxidation of PL-PUFA into PL-PUFA-OOH. Alternatively, PL-PUFA can also be oxidized to PL-PUFA-OOH by certain enzymes, such as LOXs. The resulting accumulation of peroxidized lipids subsequently triggers the execution of ferroptosis. A portion of the cytosolic iron is stored by binding to ferritin. This stored iron can be released via NCOA4-mediated ferritinophagy. By linearly ubiquitinating NCOA4, LUBAC stabilizes it and prevents its degradation, a process essential for ferritinophagy. Under conditions of elevated LUBAC activity, excessive ferritinophagy leads to iron overload, ultimately sensitizing cells to ferroptosis. This entire pathway is negatively regulated by OTULIN. The System x_c_^−^–GSH–GPX4 axis constitutes a crucial defense mechanism against ferroptosis. System x_c_^−^ exports glutamate in exchange for the import of cystine, a precursor for GSH synthesis. GPX4 utilizes GSH to reduce toxic PL-PUFA-OOH to nontoxic PL-PUFA-OH, thereby suppressing lipid peroxidation. In this process, GSH is oxidized to glutathione disulfide (GSSG), which is then reduced back to GSH by glutathione reductase (GR). Notably, the small molecules Erastin and RAS-selective lethal 3 (RSL3) are known inhibitors of System x_c_^−^ and GPX4, respectively. Furthermore, the stability and anti-ferroptotic activity of GPX4 are enhanced through its linear ubiquitination by LUBAC, a process facilitated by p21, which promotes the recruitment of the LUBAC complex.

The aforementioned studies convey a key insight: the regulatory role of linear ubiquitination in ferroptosis is not unidirectional. However, we believe that this does not represent a contradiction. It can be observed that LUBAC’s regulation of GPX4 constitutes a defensive mechanism against ferroptosis, whereas its modification of NCOA4 participates in the induction of ferroptosis. This precisely illustrates the comprehensive nature of LUBAC’s regulatory control over the ferroptosis process. In the future, it may be possible to determine whether LUBAC activates or suppresses ferroptosis by modulating specific environmental signals. Furthermore, while the regulatory outcomes of CYLD and OTULIN on NCOA4 may appear contradictory at first glance, this can be reconciled. Based on the facts that CYLD can modulate both K63 and linear ubiquitination, and that linear ubiquitin chains stabilize NCOA4, we hypothesize that CYLD may selectively remove K63-linked ubiquitin chains from NCOA4 while preserving its linear ubiquitin chains. This action would prevent NCOA4 degradation via the lysosomal pathway, thereby promoting ferroptosis. Validating this hypothesis will require further investigation. Figure 8 depicts the regulatory role of LUBAC in key ferroptosis processes.

## Linear Ubiquitination: Implications in Diverse Diseases

In this chapter, we comprehensively review the close associations between dysregulated linear ubiquitination and the pathogenesis of 5 major disease categories: neoplastic, neurodegenerative, infectious, inflammatory, and metabolic disorders, thereby providing insights to guide future research directions in these fields. For a more intuitive and comprehensive understanding of the role and regulatory mechanisms of linear ubiquitination in disease, Table 1 assembles a summary of the diseases it regulates, the key pathways/axes involved, and its critical substrate targets.

**Table 1.** Summary of diseases, key pathways/axes, and critical substrate targets in linear ubiquitination

Disease category	Associated diseases	Key pathways/axes involved	Critical substrates of linear ubiquitination
Neoplastic diseases	DLBCL	TLR–NF-κB	NEMO [107]
		BCR–NF-κB	NEMO and BCL10 [108]
	BC	TNF-α–NF-κB	NEMO [111]
		EGFR–NF-κB	NEMO [112]
		Wnt/β-catenin	β-catenin [113]
	HCC	TNF-α–NF-κB	NEMO [114]
		LUBAC–caspase-8 axis	Undefined [116]
		OTULIN–mTOR axis	Undefined [117]
		OTULIN–caspase-8 axis	Undefined [118]
	MM	TNF-α–c-FLIP axis	RIPK1 and c-FLIP [120]
	AML	LUBAC–NF-κB axis	Undefined [121]
	PCa	PI3K/AKT	PTEN [122]
	LC	LUBAC–HIF1α axis	HIF1α [123]
		LUBAC–NF-κB axis	NEMO [124]
	OS	LUBAC–NF-κB axis	Undefined [125]
	GBM	OTULIN–STAT3 axis	STAT3 [126]
	CRC	Hh	Gli [127]
		LUBAC–NF-κB axis	Undefined [128]
Neurodegenerative diseases	HD	p97–LUBAC axis	mHTT aggregates [131]
	AD	Undefined	tau NFTs [134]
	ALS	TNF-α–NF-κB	NEMO [139] and TDP-43 aggregates [141]
	PD	NEMO–p62 axis	NEMO and α-Syn aggregates [143]
Infectious diseases	*Salmonella*	RNF213–LC3 axis	*Salmonella* [144]
	*Bc*	IFNAR–LUBAC axis	*Bc* [148]
	*Mtb*	IKK–NF-κB axis	NEMO [149]
	*Lp*	TNF-α–NF-κB	LCV and NEMO [150]
	*S. flexneri*	TNF-α–NF-κB	NEMO [151]
	HBV	Parkin–MAVS axis	MAVS [152]
	HCV	TNF-α–NF-κB	NEMO [153]
	PRRSV	TNF-α–NF-κB	NEMO [154]
		OTULIN–NEMO axis	NEMO [155]
	EBV	LMP1–IRF7/NF-κB axis	NEMO and IRF7 [156]
	HTLV-1	IKK–NF-κB axis	NEMO [53]
	*T. gondii*	IFN-γ–RNF213 axis	PV [158]
Inflammatory diseases	Dermatitis	TNF-α–caspase-8 axis	RIPK1 [159]
		TNF-α–NF-κB	NEMO [160]
	Arthritis	LKB1–AMPK	LKB1 [94]
		p21–GPX4 axis	GPX4 [104]
		TRAF1–NF-κB axis	NEMO [162]
	Autoinflammatory disease	TNF-α–caspase-8 axis	LUBAC [164]
		TNF-α/IL-1β–NF-κB	NEMO [165]
	IBD	TNF-α–caspase-8 axis	RIPK1 [166]
		TLR–NF-κB/ERK	Undefined [167]
	Hepatitis	LUBAC–caspase-8 axis	Undefined [116]
		OTULIN–mTOR axis	Undefined [117]
		OTULIN–caspase-8 axis	Undefined [118]
Metabolic diseases	Type 1 DM	TNF-α–caspase-8 axis	NEMO, RIPK1, and c-FLIP [168]
	PBD	Undefined	Undefined [169,170]
	NASH	LUBAC–NF-κB axis	Undefined [171]
	Gout	TRAF1–IL-1β axis	ASC [172]

### Linear ubiquitination and neoplastic diseases

Neoplastic diseases refer to a category of disorders characterized by uncontrolled cell proliferation, leading to the formation of abnormal tissues or neoplastic lesions with invasive and metastatic potential, ultimately resulting in pathological damage. The pathogenesis of these diseases is intricately linked to linear ubiquitination, as exemplified in diffuse large B-cell lymphoma (DLBCL). DLBCL is the most common aggressive lymphoid malignancy, primarily classified into germinal center B-cell-like (GCB)-DLBCL and activated B-cell-like (ABC)-DLBCL, with the latter posing greater clinical management challenges [107]. NF-κB activation mediated by TLR and BCR signaling pathways is a distinctive feature of ABC-DLBCL. It has been demonstrated that enhanced LUBAC activity promotes the development of DLBCL in mouse models through the TLR-myeloid differentiation primary response gene 88–NF-κB pathway [107]. Within the BCR signaling pathway engaged in the pathogenesis of ABC-DLBCL, the CBM complex serves as the key trigger for the canonical NF-κB pathway. In human ABC-DLBCL cell lines, upon binding to the CBM complex, cIAP1/2 mediates K63 ubiquitination of both themselves and BCL10. The IKK complex and LUBAC are subsequently recruited and bind to the K63-linked ubiquitin chains. LUBAC then mediates the linear ubiquitination of both BCL10 and the IKK complex, leading to the activation of the downstream NF-κB pathway [108]. Targeting LUBAC activity is therefore considered a promising strategy for the treatment of DLBCL.

Breast cancer (BC) is a heterogeneous disease and the most frequent malignant tumor in women. Based on molecular and histologic evidence, BC can be divided into 3 categories: BC expressing human epidermal receptor 2, BC expressing hormone receptor (estrogen receptor [ER] or progesterone receptor), and triple-negative BC (TNBC) [109]. Relevant evidence suggests that the mRNA levels of 3 key components that make up LUBAC are notably overexpressed in human BC samples, and that the 3 components are therefore being considered as diagnostic biomarkers for BC [110]. HOIP has been recognized as a possible key drug target in ER-negative human BC [110]. Moreover, through their interaction with LUBAC, epsins promote NF-κB signaling and ER-negative BC development in mice by facilitating the recruitment of LUBAC to NEMO and augmenting NEMO’s linear ubiquitination [111]. Epidermal growth factor receptor (EGFR) can recruit and activate LUBAC via plakophilin2, thereby promoting NF-κB activation and driving the proliferation and clonogenicity of human BC cells [112]. A study involving multiple TNBC patients challenges the pro-tumorigenic role of linear ubiquitination. It is suggested that in human TNBC cells, OTULIN promotes cancer cell growth, metastasis, and drug resistance through the inhibition of β-catenin linear ubiquitination and subsequent activation of Wnt signaling [113]. Based on the aforementioned data, we conclude that the linear ubiquitination of distinct substrate proteins by LUBAC exerts divergent roles in BC. This synthesis substantially advances our mechanistic understanding of BC pathogenesis.

Liver cancer refers to malignant tumors of the liver, which can be classified into primary and secondary types. Hepatocellular carcinoma (HCC) is the most common form of primary liver cancer, characterized by the malignant transformation and uncontrolled proliferation of hepatocytes. For HCC patients, HOIP promotes cancer cell proliferation and invasion by regulating TNF-α-induced NF-κB activation [114]. HOIL-1L stabilizes HOIP by inhibiting its ubiquitin-mediated degradation, thereby promoting the proliferation and metastasis of human HCC cells [115]. However, findings from other scholars have revealed that LUBAC acts as a tumor suppressor in HCC [116]. Deficiency in LUBAC activity induces caspase-dependent apoptosis and hepatitis, followed by compensatory cell proliferation, with the final outcome being HCC development in mice [116]. We propose that these contradictory findings may be attributed to species-specific differences, which warrants further investigation. Additionally, OTULIN deficiency leads to severe steatohepatitis in mice accompanied by aberrant mechanistic target of rapamycin (mTOR) activation. The increased mTOR activity not only exacerbates hepatic fibrosis but also promotes the development of HCC. Dysregulated linear ubiquitination resulting from loss of OTULIN function serves as a key driver for these pathological processes [117]. Another study confirms the critical role of OTULIN-maintained linear ubiquitination homeostasis in protecting against hepatocyte apoptosis and hepatitis, demonstrating that OTULIN deficiency triggers FADD- and RIPK1 kinase activity-dependent apoptosis, which subsequently induces compensatory hepatocyte proliferation and ultimately drives HCC development in mice [118]. In conclusion, both LUBAC and OTULIN serve as critical regulators of hepatic homeostasis, whose proper functioning constitutes an essential requirement for protection against HCC development. However, species-specific differences must be carefully considered in studies investigating the relationship between linear ubiquitination and HCC.

Additionally, linear ubiquitination affects the process of tumorigenesis and progression in many other types of tumors. There are 3 main types of skin cancer: melanoma (MM), squamous cell carcinoma (SCC), and basal cell carcinoma. Studies have confirmed that the level of linear ubiquitination is markedly enhanced in human SCC cells and mouse MM cells [119]. Research suggests that loss-of-function mutations in PP6 may promote the development of murine MM cells by sustaining Met1 ubiquitination of RIPK1 and c-FLIP [120]. Furthermore, HOIP has been demonstrated to be essential for maintaining the proliferation levels of both mouse and human acute myeloid leukemia (AML) cells, and inhibiting LUBAC activity may represent an effective therapeutic strategy for AML in the future [121]. The linear ubiquitination of phosphatase and tensin homolog (PTEN) leads to a pronounced loss of its activity, a process that enhances phosphoinositide 3-kinase (PI3K)/protein kinase B (AKT) signaling, thereby driving the progression of prostate cancer (PCa) in patients and potentially increasing the risk of recurrence [122]. Notably, LUBAC can enhance the stability and activity of hypoxia-inducible factor-1α (HIF1α) by depositing Met1-linked ubiquitin chains onto it, thereby counteracting its degradation by CMA and ultimately promoting the proliferation, invasion, and migration of human lung cancer (LC) cells [123]. Lung SCC (LSCC) is one of the most common subtypes of LC. LUBAC is a determinant of cisplatin resistance in both human and mouse LSCC. Inhibiting LUBAC activity to block the NF-κB pathway is key to resensitizing LSCC-bearing mice to cisplatin [124]. At the same time, NF-κB activation mediated by LUBAC activity is an important induction condition for murine osteosarcoma (OS) cells to invade tissues and develop lung metastasis [125]. It has been found that signal transducer and activator of transcription 3 (STAT3), following its linear ubiquitination, is dephosphorylated by T-cell protein Tyr phosphatase, which consequently prevents its activation and nuclear translocation [126]. Conversely, OTULIN maintains persistent STAT3 activation by removing the Met1-linked ubiquitin chains from STAT3, which, in turn, drives the stemness and self-renewal of human GBM cells [126]. Furthermore, by stabilizing glioma-associated oncogene (Gli) via linear ubiquitination, LUBAC promotes noncanonical activation of the Hedgehog (Hh) signaling, a process that drives the proliferation of cancer cells in CRC patients [127]. Cyclophilin J (CYPJ) is an important tumor suppressor that restricts LUBAC-mediated NF-κB activation by impeding the interaction between HOIP and SHARPIN, thereby inhibiting the proliferation and migration of human CRC cells [128].

Currently, most research findings characterize LUBAC as a tumor promoter, and many tumorigenic mechanisms involve LUBAC-mediated activation of NF-κB signaling. Thus, designing antitumor drugs targeting linear ubiquitination and NF-κB may represent an important future strategy for cancer treatment. However, since some scholars still point out that linear ubiquitination has a clear tumor-suppressive effect in certain types of tumors, the relationship between linear ubiquitination and tumors still deserves to be further explored.

### Linear ubiquitination and neurodegenerative diseases

Neurodegenerative disease is an umbrella term for a range of conditions characterized by progressive loss of neuronal structure and function, and representative disorders include Huntington’s disease (HD), Alzheimer’s disease (AD), amyotrophic lateral sclerosis (ALS), and Parkinson’s disease (PD). A hallmark pathological feature of neurodegenerative diseases is the abnormal deposition of misfolded protein aggregates [129]. Also, protein aggregates are important pathogenic factors in neurodegenerative diseases, and linear ubiquitination is involved in the formation, sequestration, and degradation of misfolded protein aggregates to intervene in the course of neurodegenerative diseases through multiple pathways.

HD is a multifactorial triggered neurodegenerative disease, and excessive intracellular production and aggregation of mutant huntingtin (mHTT) is an important feature of HD. It has been established that mHTT aggregates colocalize with linear ubiquitin chains in HeLa cells, and that these chains on mHTT serve as a critical requirement for OPTN recruitment. When the linear ubiquitin-binding capacity of OPTN is impaired, it may disrupt the autophagic clearance of mHTT and lead to its accumulation [130]. Additionally, LUBAC is recruited to mHTT aggregates in a p97-dependent manner. This recruitment induces linear ubiquitination of the aggregates, promoting their degradation through the proteasomal pathway and thereby ameliorating mHTT-induced toxicity in both human and mouse cells [131]. Therefore, how to increase the degree of linear ubiquitination of mHTT aggregates may be an important entry point for the future treatment of HD.

AD is one of the most common dementia-inducing neurological disorders today. Excessive accumulation of characteristic amyloid plaques formed by oligomerization of amyloid-beta (Aβ) from amyloid precursor protein hydrolysis is a key pathogenetic mechanism of AD. A clinical study revealed that Aβ aggregation in patient macrophages induces a reactive up-regulation of SHARPIN, which, in turn, mediates the phagocytosis of Aβ plaques by promoting the expression of phagocytic receptors [132]. Moreover, it has been proposed that all pathway mechanisms involved in Aβ clearance and degradation are transcriptionally orchestrated by NF-κB, which, in turn, is potently regulated by the LUBAC-dependent SHARPIN signaling [133]. While this theory strengthens the mechanistic link between LUBAC and Aβ pathology, it is evident that NF-κB is not exclusively regulated by LUBAC. Further research and evidence are still required to substantiate the hypothesis that LUBAC modulates Aβ clearance and degradation through NF-κB. Neurofibrillary tangles (NFTs) formed by tau protein oligomers are another prominent histopathological hallmark of AD. Although linear ubiquitin chains have been demonstrated to colocalize with tau NFTs in the brains of AD patients [134], the specific regulatory role of LUBAC in tau NFT pathology remains to be further elucidated. Notably, aberrant LUBAC activity has been implicated in AD, and single-nucleotide polymorphisms (SNPs) in SHARPIN and HOIL-1L have recently been identified as genetic risk factors for AD [134]. Collectively, this evidence suggests that LUBAC may contribute to AD pathogenesis by modulating the accumulation of both Aβ and tau.

ALS is a fatal neurodegenerative disease characterized by progressive loss of motor neurons in the brain and spinal cord. Nuclear clearance and aberrant cytoplasmic aggregation of TAR DNA-binding protein 43 (TDP-43) is a common pathological feature seen in neurons of most ALS patients [135]. Available evidence suggests that TDP-43 is primarily degraded by the ubiquitin–proteasomal system, whereas the degradation of TDP-43 aggregates is dependent on autophagy [136]. The autophagy receptor OPTN suppresses TDP-43 aggregation by mediating its autophagic degradation. However, the E50K mutation in OPTN impairs this function, leading to the accumulation of TDP-43 aggregates in mouse retinal ganglion cells [137]. In Neuro2a cells, binding to linear ubiquitin chains is a prerequisite for OPTN to dissociate from Ras-related protein Rab-8A (Rab8A) and subsequently bind microtubule-associated protein 1 light chain 3 (LC3), thereby activating autophagy [138]. It is noteworthy that ALS-associated mutations in human OPTN impair its ability to bind linear ubiquitin chains. This defect leads to dysregulated IKK-mediated NF-κB activation, which constitutes a key mechanism in ALS pathogenesis [139]. A study of 12 ALS patients demonstrated the colocalization of TDP-43 aggregates with linear ubiquitin chains, HOIP, and SHARPIN in neuronal cells. The authors further observed that OPTN is recruited to TDP-43 aggregates enriched in linear ubiquitin, leading them to hypothesize that linear ubiquitination may facilitate the clearance of pathological TDP-43 via the autophagy pathway [140]. Intriguingly, a contrary conclusion has been reported, showing that in Neuro2a cells, the association of linear ubiquitin chains with TDP-43 promoted the formation of insoluble aggregates. Moreover, inhibition of LUBAC activity not only considerably reduced cytosolic TDP-43 aggregation but also down-regulated TNF-α-mediated NF-κB activation [141]. In conclusion, influencing the production, aggregation, and elimination of TDP-43 by manipulating linear ubiquitination will be an interesting direction for future research on ALS.

PD is a progressive neurodegenerative disease characterized by movement disorders, and its mainly pathological characteristics are the loss of dopamine neurons in the substantia nigra pars compacta and the formation of large numbers of α-synuclein (α-Syn)-rich Lewy bodies [142]. In both human and mouse cells, it has been established that NEMO promotes the recruitment of p62 to inclusions by amplifying linear ubiquitination signals on α-Syn aggregates and binding to Met1-linked ubiquitin chains. This process facilitates the autophagic clearance of protein aggregates, representing a key mechanism through which NEMO maintains proteostasis [143]. Therefore, findings from these studies demonstrate that modulating linear ubiquitination to preserve protein homeostasis in neurons is a viable strategy for future PD treatment.

A strong link exists between linear ubiquitination and various types of misfolded protein aggregates. Consequently, developing drugs that target the linear ubiquitination of these aggregates represents a promising therapeutic strategy for the future treatment, and potentially the cure, of neurodegenerative diseases.

### Linear ubiquitination and infectious diseases

Infectious diseases are disorders caused by pathogenic microorganisms that invade the body, proliferate within it, and disrupt normal physiological functions. Linear ubiquitination is involved in the development and progression of infectious diseases through various pathways. Among these, the relationship between *Salmonella* and linear ubiquitination is particularly close. *Salmonella* infection mainly causes gastroenteritis, typhoid fever, and septicemia. When *Salmonella* invades human and mouse cells, the host cell activates the xenophagy pathway to eliminate the bacteria. During this process, LUBAC deposits linear ubiquitin chains on the surface of *Salmonella*, which recruits the autophagy receptor OPTN to bind the ubiquitin chains, thereby activating xenophagy [144]. Impaired LUBAC activity, such as in HOIP knockout, suppresses the initiation of xenophagy, leading to the death of *Salmonella*-infected human cells [145]. On the other hand, genetic defects in autophagy receptors like nuclear dot protein 52 markedly reduce the linear ubiquitination of *Salmonella*, severely impairing the formation of xenophagosomes and bacterial clearance in human cells [146]. Furthermore, studies have revealed that depletion of OTULIN enhances the formation of Met1-linked ubiquitin chains on *Salmonella*, leading to localized recruitment of NEMO and subsequent activation of NF-κB. The resulting amplification of NF-κB-dependent inflammatory cytokine secretion further restricts bacterial replication in HeLa cells [147]. Thus, LUBAC not only clears invading *Salmonella* via the xenophagy pathway but also suppresses bacterial proliferation through NF-κB activation. However, it remains to be elucidated whether other consequences resulting from loss of OTULIN function may exert negative effects during the later stages of the cellular antibacterial response. *Burkholderia cenocepacia* (*Bc*) is commonly associated with lung infections in patients with cystic fibrosis, chronic granulomatous disease, and other immunodeficiencies. LUBAC deposits linear ubiquitin chains onto the surface of *Bc* that invade human and mouse macrophages, thereby facilitating the degradation and disruption of the bacteria via the autophagy pathway [148]. Moreover, when *Bc* escape into the host cell cytosol, type I IFN signaling up-regulates the components of LUBAC, which is a key driver for the subsequent cell-autonomous anti-*Bc* immune response [148]. Tuberculosis, caused by *Mycobacterium tuberculosis* (*Mtb*) infection, remains a global public health threat. Following the invasion of THP-1 cells by *Mtb*, its effector PPE60 promotes the expression of pro-inflammatory cytokines and alters host cell fate through LUBAC-mediated NF-κB signaling [149]. *Legionella pneumophila* (*Lp*) primarily causes respiratory diseases in humans, such as legionellosis. RavD is an effector protein encoded by *Lp* that specifically hydrolyzes linear ubiquitin chains. By preventing the accumulation of linear ubiquitin chains on the *Lp*-containing vacuole (LCV), RavD suppresses the activation of the TNF-α–NF-κB pathway during infection of human cells by *Lp* [150]. *Shigella flexneri* is an enteroinvasive pathogen that causes more than one million deaths annually. Upon infecting human cells, *S. flexneri* secretes 2 effector proteins, IpaH1.4 and IpaH2.5, which directly interact with HOIL-1L and HOIP. These effectors subsequently catalyze the conjugation of K48-linked ubiquitin chains to the RBR domain of HOIP, thereby promoting its degradation via the proteasomal pathway. This process ultimately leads to irreversible inactivation of the LUBAC and suppression of NF-κB nuclear translocation [151]. It can thus be concluded that inhibiting the innate immune response of cells to infection by inducing LUBAC dysfunction is a core immune evasion strategy of *Lp* and *S. flexneri*.

In addition to bacteria, viruses can also influence innate immune signaling of the body by hijacking linear ubiquitination. Chronic infection of hepatitis B virus (HBV) and hepatitis C virus (HCV) is one of the main causes of HCC. Upon invading human cells, HBV utilizes Parkin to recruit the LUBAC to mitochondria, where LUBAC-mediated linear ubiquitination of mitochondrial antiviral signaling protein (MAVS) disrupts the MAVS signalosome and hinders downstream IFN synthesis [152]. In vitro studies demonstrate that HCV facilitates the direct binding of NS3 to LUBAC, which competitively blocks the interaction between LUBAC and NEMO, thereby suppressing the linear ubiquitination of NEMO and subsequent NF-κB activation in human cells [153]. Porcine reproductive and respiratory syndrome virus (PRRSV) has always been considered one of the most important swine pathogens affecting the global swine industry. Nonstructural protein 1α encoded by PRRSV binds to HOIP and HOIL-1L, thereby disrupting the interaction between HOIP and SHARPIN. This disruption restricts the LUBAC-dependent linear ubiquitination of NEMO, thereby blocking NF-κB signaling in virus-infected HEK293T and MARC-145 cells [154]. In both human and porcine cells, PRRSV not only up-regulates OTULIN gene expression but also utilizes its encoded nonstructural protein 11 to recruit OTULIN and enhance its ability to remove linear ubiquitin chains from NEMO, thereby suppressing IFN production [155]. Epstein–Barr virus (EBV) is an oncogenic virus associated with a variety of lymphoid malignancies. In both human and mouse cells, EBV employs its encoded latent membrane protein 1 (LMP1) to differentially regulate the activities of IRF7 and NF-κB. During this process, LMP1-mediated activation of the NF-κB pathway requires linear ubiquitination of NEMO, whereas linear ubiquitination of IRF7 by LUBAC substantially suppresses IRF7 activity, thereby inhibiting IFN-β production mediated by the LMP1–IRF7 signaling axis [156]. EBV-positive DLBCL represents a distinct subtype of DLBCL, characterized by constitutive NF-κB activation as a key hallmark. The critical role of EBV-mediated NF-κB signaling in the pathogenesis observed in patients has been consistently emphasized [157]. Thus, EBV-induced hyperactivation of the LUBAC-dependent LMP1–NF-κB pathway may be a key mechanism underlying DLBCL development. HTLV-1 is an oncogenic retrovirus that causes adult T-cell leukemia (ATL). The HTLV-1-encoded Tax protein recruits LUBAC to the IKK complex, inducing the formation of K63/Met1-linked hybrid ubiquitin chains in human and mouse cells. This process establishes persistent NF-κB activation, which may represent a principal mechanism underlying HTLV-1-induced ATL [53]. Therefore, sometimes, LUBAC can also be exploited by certain viruses as a tool to maintain their own long-term survival.

In addition to bacteria and viruses, host immune responses to protozoan infections are modulated by linear ubiquitination. *Toxoplasma gondii* is an obligate intracellular protozoan parasite that causes toxoplasmosis, a zoonotic disease. Upon invading human cells, *T. gondii* triggers the production of IFN-γ, which, in turn, induces the translocation of ring finger protein 213 (RNF213) to the *T. gondii*-containing parasitophorous vacuole (PV). RNF213 facilitates the formation of K63- and Met1-linked ubiquitin chains on the PV membrane, leading to the recruitment of host defense proteins and the triggering of anti-parasitic immune responses [158]. Intriguingly, the authors note that LUBAC appears to be dispensable for the ubiquitination of PV and for cell-autonomous host defense, a phenomenon that warrants further investigation [158].

In summary, LUBAC participates in modulating immune responses against pathogens through multiple mechanisms. Whether LUBAC functions properly is critical for pathogen survival, sustained infection establishment, and tumorigenesis induction.

### Linear ubiquitination and inflammatory diseases

Inflammatory diseases are a group of disorders characterized by inflammation as the core pathological process and essential feature, and they represent the category most markedly subject to regulation by linear ubiquitination. Dermatitis is a typical inflammatory disease, characterized by impaired epidermal barrier function and immune cell infiltration. Some scholars have pointed out that LUBAC activity is crucial for maintaining skin homeostasis. LUBAC dysfunction drives the downstream signaling pathways mediated by TNF-α, TRAIL, and FasL, promoting excessive apoptosis and ultimately leading to lethal dermatitis in mice [159]. Some researchers have emphasized that the deficiency of SHARPIN substantially reduces the amount of HOIP and HOIL-1L, leading to chronic proliferative dermatitis in mice (cpdm) [160]. They also demonstrated that IFN-γ and/or IFN-α enhance NF-κB activation and suppress TNF-α-mediated apoptosis in mouse cells by up-regulating HOIP and HOIL-1L expression and increasing LUBAC levels, thereby ameliorating cpdm [160]. For OTULIN, its mediated linear deubiquitination of proteins has been confirmed to be essential for maintaining skin homeostasis, preventing keratinocyte death, and suppressing skin inflammation. Once OTULIN is ablated, it leads to the death of keratinocytes and subsequently to skin inflammation and verrucous carcinoma in mice [161]. Thus, it can be concluded that the dynamic homeostasis of linear ubiquitination maintained by LUBAC and OTULIN is critical for preserving skin homeostasis and protecting against the development of dermatitis.

Arthritis is an inflammatory disease that occurs in the joints and their surrounding tissues. Linear ubiquitination of LKB1 enhances its activity and activates AMPK, which, in turn, suppresses the NLRP3 inflammasome response, thereby reducing pyroptosis in human chondrocytes [94]. Based on this evidence, the authors emphasize that promoting linear ubiquitination of LKB1 represents a potential therapeutic strategy for OA [94]. Ferroptosis is also an important participant in the pathogenesis of OA. The high expression of p21 in mouse chondrocytes drives linear ubiquitination of GPX4 by promoting the LUBAC–GPX4 interaction. This linear ubiquitination serves as a mechanism to stabilize GPX4, thereby impeding ferroptosis and conferring resistance to OA in mice [104]. Interestingly, evidence indicates that in human cells, TNFR-associated factor 1 (TRAF1) binds to the LUBAC and impairs linear ubiquitination of NEMO, thereby restricting TLR signaling [162]. However, a rheumatoid arthritis (RA)-associated SNP in the human TRAF1 gene disrupts this restriction on TLR signaling and enhances the production of inflammatory cytokines, providing a compelling explanation for the increased incidence and severity of RA and other inflammatory diseases [162]. In summary, focusing on the critical role of linear ubiquitination in the pathogenesis of arthritis may lead to unforeseen therapeutic benefits for this disease.

Autoinflammatory disease is a collective term for a series of disorders characterized by recurring episodes of inflammation due to specific defects in the innate immune system. In both humans and mice, loss-of-function mutations in OTULIN lead to the development of an autoinflammatory disease known as OTULIN-related autoinflammatory syndrome (ORAS). Patients with ORAS exhibit substantial accumulation of linear ubiquitin chains in their leukocytes, similarly observed in mouse BMDMs [163]. Notably, anti-TNF therapy is generally effective in alleviating ORAS symptoms in both species [163]. A study involving patients with ORAS has elucidated part of its pathogenesis: mutations in the OTULIN gene lead to compromised catalytic activity, while LUBAC function is weakened due to its excessive auto-linear ubiquitination. This promotes the formation of cell death-inducing complex II and enhances TNF-α-mediated cell death. The large number of dying cells release DAMPs and other pro-inflammatory factors, thereby eliciting inflammatory responses [164]. HOIL-1L deficiency leads to reduced protein levels of SHARPIN and HOIP, resulting in a subsequent impairment in LUBAC complex assembly. These findings are supported by a study of 3 HOIL-1-deficient patients, which demonstrated impaired LUBAC-dependent NF-κB pathway activation in patient-derived fibroblasts. The patients presented with marked immunodeficiency and chronic autoinflammation, the latter of which may result from exaggerated leukocyte responses to IL-1β [165]. We propose that developing agonists targeting LUBAC or OTULIN will offer therapeutic promise for patients with autoinflammatory diseases.

Inflammatory bowel disease (IBD) is a group of chronic nonspecific inflammatory diseases of the intestines of undetermined etiology. Researchers have proposed that A20 overexpression is a critical factor in the pathogenesis of IBD. They discovered that overexpressed A20, after homodimerizing, binds to linear ubiquitin chains on RIPK1. This interaction promotes the assembly and stability of the ripoptosome complex, leading to enhanced recruitment and activation of caspase-8. Ultimately, this results in extensive TNF-α-induced, RIPK1-dependent death of mouse intestinal epithelial cells (IECs) [166]. Furthermore, in mouse models, LUBAC dysfunction in IECs not only triggers intestinal inflammation but also sensitizes these cells to TNF-mediated cell death. In contrast, loss of LUBAC activity in macrophages considerably attenuates dextran sulfate sodium (DSS)-induced colitis and impairs the TLR-driven activation of NF-κB and extracellular signal-regulated kinase (ERK) along with the subsequent inflammatory response [167]. These findings demonstrate that linear ubiquitination in IECs and macrophages plays different roles in the progression of IBD, and that cell-specific targeting of linear ubiquitination might be a novel therapeutic approach for IBD.

Hepatitis is defined as an inflammatory response in the liver caused by diverse pathogenic factors and is characterized by hepatocellular damage and concomitant abnormalities in liver function. Currently, chronic hepatitis is considered to be the main cause of HCC. Dysfunction of LUBAC in hepatocytes promotes cell apoptosis and disrupts hepatic homeostasis. The subsequent increase in hepatocyte death leads to TNFR1-mediated inflammation, compensatory hepatocyte proliferation, and DNA damage. This tumor-promoting environment ultimately drives the development of HCC in mice [116]. However, other researchers have revealed that hepatocyte-specific OTULIN deficiency in mice triggers steatohepatitis, hepatic fibrosis, and tumorigenesis. This pathological process occurs independently of TNFR1 signaling transduction, but is mechanistically linked to dysregulated linear ubiquitination and aberrant mTOR activation [117]. Further investigation revealed that the TNFR1-independent cell death pathway in OTULIN-deficient mice depends on the kinase activities of both FADD and RIPK1. Furthermore, the authors demonstrated that OTULIN confers protection against liver inflammation and HCC development through its precise cleavage of Met1-linked ubiquitin chains and subsequent suppression of hepatocyte apoptosis [118]. Based on the above evidence, it can be established that LUBAC deficiency and OTULIN dysfunction induce distinct patterns of linear ubiquitination dysregulation. These 2 defects promote murine hepatitis and HCC development through TNFR1-dependent and TNFR1-independent pathways, respectively.

It is evident that linear ubiquitination is closely associated with a wide range of inflammatory diseases. Dysregulation of linear ubiquitination often leads to immunodeficiency and cell death, and the resulting chronic inflammatory environment can further promote the development of various tumors. In addition, the cell-type-specific role of linear ubiquitination is also not negligible in related studies. Therefore, maintaining the stability of LUBAC’s catalytic activity represents one of the key future strategies for managing inflammatory diseases.

### Linear ubiquitination and metabolic diseases

Beyond the aforementioned diseases, growing research highlights the pivotal regulatory role of linear ubiquitination in the pathogenesis of metabolic diseases, disorders marked by aberrant metabolism of substances such as sugars, lipids, and proteins. It is well-established that diabetes mellitus (DM) constitutes a prototypical and severe metabolic disease. It has been confirmed that N-acetylcysteine combined with insulin protects against excessive apoptosis in canine cardiomyocytes in a type 1 DM model. This protection is mediated by modulating the linear ubiquitination of NEMO, RIPK1, and c-FLIP within the TNF-α-mediated extrinsic apoptosis pathway [168]. This study provides a new therapeutic direction for alleviating cardiac complications of type 1 DM. Polyglucosan body disease (PBD) is a group of inherited metabolic disorders caused by impaired glycogen metabolism, and myopathy is often a complication of PBD. Notably, LUBAC has recently been identified as a key player in the pathogenesis of PBD [169]. Mutations in HOIL-1L have been suggested as a common trigger of polyglucosan body myopathy (PGBM). A study involving 4 patients with PGBM revealed that missense or truncating mutations in the HOIL-1L gene caused substantial polyglucosan accumulation in cardiac and skeletal muscles, thereby promoting the development of progressive muscle weakness and cardiomyopathy in these patients [170]. The A18P mutation in HOIL-1L disrupts the assembly of the LUBAC complex and induces the formation of aberrant glycogen [169]. In addition to HOIL-1L, patients with HOIP deficiency also exhibit impaired LUBAC assembly concomitant with abnormal glycogen accumulation in lower limb muscles [169]. The authors further concluded that the formation of the LUBAC complex, even in limited amounts, is adequate to prevent the onset of immune disorders but inadequate to avert PBD [169]. To date, research exploring the relationship between linear ubiquitination and glycogen metabolism remains remarkably limited. Enhancing investigations into the regulatory role of linear ubiquitination in glycogen metabolic disorders may open new therapeutic avenues for clinical patients.

Lipid metabolism and uric acid metabolism are also strongly related to linear ubiquitination. In a mouse model of nonalcoholic steatohepatitis (NASH), hepatic fatty acid accumulation likely disrupts LUBAC assembly by reducing SHARPIN expression, thereby impairing downstream NF-κB activation [171]. Importantly, compromised NF-κB signaling in hepatocytes serves as a critical factor driving steatohepatitis and HCC development [171]. Hence, therapeutic modulation of LUBAC activity in NASH patients may yield beneficial outcomes. Gout is an inflammatory arthritis caused by disturbances in uric acid metabolism in the body. TRAF1 is a critical signaling adaptor protein that, in THP-1 cells, restricts the linear ubiquitination and oligomerization of ASC to prevent inflammasome activation and subsequent IL-1β secretion [172]. Moreover, genetic knockout of TRAF1 markedly exacerbates joint inflammation and swelling in a murine gout model [172]. The authors therefore suggest that this unique inhibition of inflammasome activation by TRAF1 is important for limiting the onset and progression of gout. It is evident that linear ubiquitination is implicated in the regulation of both lipid and uric acid metabolism. However, it exerts contrasting roles in associated pathologies: LUBAC activity suppresses NASH progression but conversely drives gout pathogenesis. Therefore, its functions must be assessed with reference to the specific disease context.

Obviously, the correct linear ubiquitination of specific target proteins ensures normal metabolic homeostasis and is a critical determinant for preventing metabolic diseases. Nevertheless, mechanistic links between linear ubiquitination and metabolic diseases remain underexplored. Future research elucidating their interplay is anticipated to yield novel clinical benefits for patients.

## Conclusion and Future Perspectives

Precise cell fate determination is fundamental to development from single cells to complex organisms and represents a critical target for maintaining health and treating disease. Dysregulation of this process leads to uncontrolled cellular states, organ dysfunction, and disrupted compensatory mechanisms, posing substantial risks to organismal health. Linear ubiquitination, a recently characterized PTM, has emerged as a key regulatory mechanism in these processes. It exerts indispensable functions in cellular turnover, metabolism, immune regulation, and tissue homeostasis by specifically controlling core biological processes including cell proliferation, differentiation, senescence, and survival/death.

The intricate relationship between linear ubiquitination and cell fate determination provides substantial conceptual insights. First, the dual role of LUBAC activity: In organs composed of terminally differentiated cells such as cardiomyocytes, the pro-survival effects of LUBAC are essential for maintaining normal organ function, whereas in tumor cells, these same pro-survival effects become drivers of tumor initiation, progression, and metastasis. Second, the potential cell- and species-specific functions of LUBAC: In the context of necroptosis, while all studies using murine cells support an inhibitory role for LUBAC, research in human cells demonstrates that LUBAC either has no effect or even promotes necroptosis. This discrepancy warrants serious consideration, as it indicates that findings from animal studies or specific cellular models cannot be indiscriminately extrapolated to human systems or other cell types in LUBAC research. Third, the comprehensive regulatory capacity of LUBAC: In both cell senescence and ferroptosis, LUBAC simultaneously regulates their initiation mechanisms and cellular defense pathways. This functional duality reflects profound regulatory depth rather than mechanistic contradiction, underscoring the necessity for precise modulation of LUBAC activity when developing interventions targeting these physiological processes. Fourth, differential effects of LUBAC deficiency versus OTULIN impairment: Studies in human cells have confirmed that GPX4 utilizes LUBAC-mediated linear ubiquitination to stabilize itself [103]. However, other researchers have demonstrated that in human OS cells, OTULIN deficiency-induced increase in GPX4 linear ubiquitination fails to enhance GPX4 protein stability [173]. Building upon this intriguing observation, we postulate that, beyond cell-type-specific effects, the distinct intracellular milieu resulting from LUBAC deficiency versus OTULIN dysfunction may represent another critical determinant. Validating this hypothesis would substantially advance research in this field. Fifth, the growing significance of LUBAC activity in human diseases: While the study of PTMs has been central to understanding human disease mechanisms, the role of linear ubiquitination has remained relatively underappreciated. This review synthesizes growing evidence that establishes its critical regulatory functions across a range of diseases. Indeed, neoplastic, neurodegenerative, infectious, inflammatory, and metabolic disorders are all influenced to varying degrees by linear ubiquitination. Consequently, targeting linear ubiquitination has emerged as a promising direction for elucidating the pathogenesis of major human diseases and developing precision therapeutic strategies.

However, at the same time, this review still has some limitations: linear ubiquitination is not the only determinant of cell fate regulation; the effects of the LUBAC-independent functions of the various components of LUBAC on cell fate determination need to be further clarified; the specific mechanism by which linear ubiquitination regulates cell fate determination and the deep relationship between the two still need to be explored in depth. In the future, we hope that through the integration of multidisciplinary tools, interdisciplinary collaborations, and the construction of appropriate animal disease models, researchers will promote the in-depth analysis of the molecular mechanisms in this field, thereby enhancing the translational potential of linear ubiquitination in the treatment of clinical diseases.

Given the critical role of linear ubiquitination in various diseases, a range of agonists (e.g., troglitazone) and antagonists (e.g., HOIP inhibitor-1/8 [HOIPIN-1/8]) capable of modulating this process have been developed [174]. Table 2 summarizes potential candidate therapeutic agents that modulate the linear ubiquitination process and elucidates their respective mechanism of action. However, the development of drugs targeting linear ubiquitination continues to face formidable challenges. First, difficulties in detection and analysis: the low abundance and highly dynamic nature of linear ubiquitination make it difficult to capture stably; the high abundance of other types of ubiquitin chains causes severe interference in detection; and linear ubiquitination targets vary across different cell types. Second, technical limitations: drug target development technologies, such as proteolysis-targeting chimera, carry the risk of off-target effects; the enrichment efficiency in proteomics remains suboptimal; and tools for resolving cell-type-specific linear ubiquitination targets are lacking. Third, additional obstacles: interpatient variability and the inability of experimental models to fully recapitulate the complex human microenvironment. Nonetheless, with the continuous advancement of science and technology, we believe that these difficulties will eventually be overcome. In conclusion, with the development of medicine, the precise regulation of cell fate through mediating linear ubiquitination may become the driving force of the medical revolution, which will ultimately provide brand-new possibilities for the prolongation of human lifespan, the improvement of the quality of life, and the cure of related diseases.

**Table 2.** A collection of agents that modulate the linear ubiquitination process and their mechanism of action

Agent class	Agent name	Mechanism of action
LUBAC antagonist	Gliotoxin [149]	Selective inhibition of LUBAC via binding to the RBR domain of HOIP
	HOIPIN-1/8 [174]	Inhibition of the RING-HECT hybrid reaction in HOIP via modification of the active site Cys885
	Aureothricin [107]	Undefined
	Thiolutin [121]	Direct inhibition of the HOIP RBR domain
	Compound (5)/(11a) [174]	Prevention of ubiquitin loading onto the HOIP RBR domain
	BAY 11-7082 [175]	Blockade of ubiquitin binding to ubiquitin-conjugating enzyme H7 and its transfer to the HOIP active site
	Bendamustine [174]	Direct HOIP binding leading to inhibition of its function
	α-helical stapled SHARPIN peptides [15]	Inhibition of LTM-mediated HOIL-1L/SHARPIN heterodimerization
	HOIP-based stapled α-helical peptides [174]	Disruption of the HOIL-1L–HOIP interaction
	Single-domain antibodies [174]	Binding to the HOIP RBR domain leading to inhibition of its function
LUBAC agonist	IFN-γ and IFN-α [160]	Induction of HOIP and HOIL-1L mRNA transcription and increase in LUBAC levels
OTULIN antagonist	PR619 [176]	Undefined
OTULIN agonist	N/A	
CYLD antagonist	Subquinocin [177]	Interaction with 2 Tyr residues in the CYLD catalytic groove leading to inhibition of its activity
CYLD agonist	Troglitazone [178]	Induction of CYLD promoter transactivation
SPATA2 antagonist	N/A	
SPATA2 agonist	N/A	

N/A, not applicable
